# Biochemical analysis of the TPS-a subfamily in *Medicago truncatula*

**DOI:** 10.3389/fpls.2024.1349009

**Published:** 2024-02-15

**Authors:** Hannah Hendrickson, Monirul Islam, Ghislain Fotso Wabo, Sibongile Mafu

**Affiliations:** ^1^ Plant Biology Graduate Program, University of Massachusetts-Amherst, Amherst, MA, United States; ^2^ Department of Biochemistry and Molecular Biology, University of Massachusetts-Amherst, Life Science Laboratories, Amherst, MA, United States; ^3^ Department of Organic Chemistry, University of Yaoundé 1, Yaounde, Cameroon

**Keywords:** *Medicago truncatula*, terpene synthase, sesquiterpenes, biochemical analysis, chemical diversity

## Abstract

Terpenes are important mediators of plant chemical response to environmental cues. Here, we describe the genome-wide identification and biochemical characterization of TPS-a members in *Medicago truncatula*, a model legume crop. Genome mining identified thirty-nine full-length terpene synthases with a significant number predicted to produce monoterpenes and sesquiterpenes. Biochemical characterization of the TPS-a subfamily associated with sesquiterpene biosynthesis revealed such compounds, that exhibit substantial biological activity in other plants. Gene expression analysis using qPCR and the *Medicago* gene atlas illustrated distinct tissue and time-based variation in expression in leaves and roots. Together our work establishes the gene-to-metabolite relationships for sesquiterpene synthases in *M. truncatula*. Understanding the biosynthetic capacity is a foundational step to defining the ecological roles of this important family of compounds.

## Introduction

**Figure 1:**
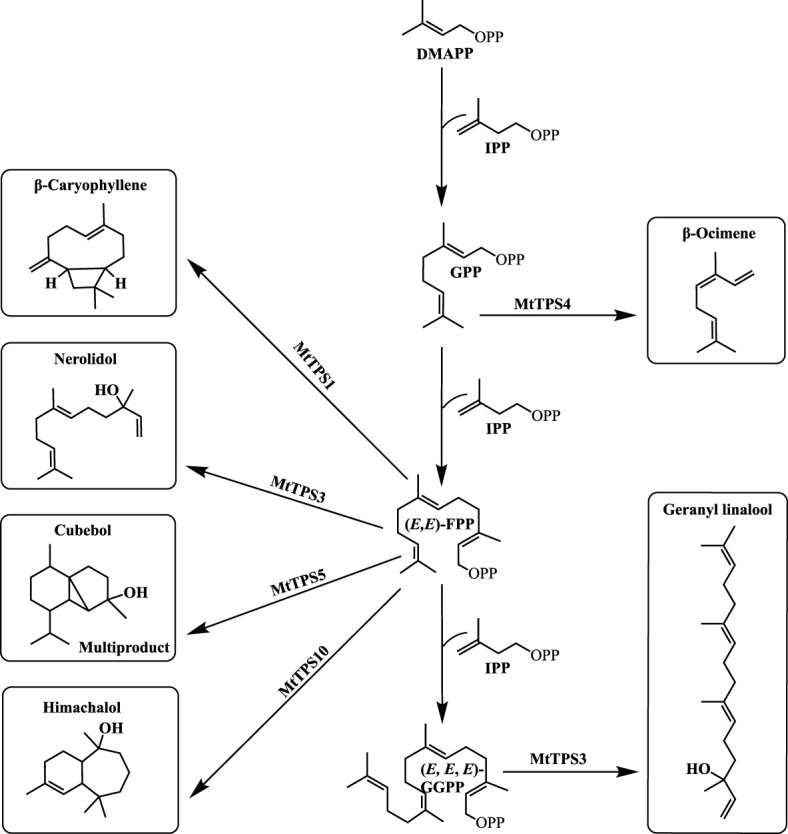
Schematic of terpene biosynthesis. Three major substrates are used in the production of most terpene products by TPSs: GPP (C10), (*E, E*)-FPP (C15), and (*E, E, E*)-GGPP (C20). Resultant terpene products of previously characterized *Mt*TPSs: *Mt*TPS1 produces β-caryophyllene, *Mt*TPS3 produces both the sesquiterpene product nerolidol and the diterpene product geranyl linalool, *Mt*TPS4 produces β-ocimene, *Mt*TPS5 produces cubebol as a major product but is a multiproduct enzyme and *Mt*TPS10 produces mainly himachalol.

Plants produce an array of specialized metabolites in response to environmental stressors. Terpenoids are one such class of natural products with unprecedented chemical diversity. Biosynthesis proceeds from simple core steps which initiate from five-carbon units to make varying lengths of backbones (**Figure 1**). Terpene synthases (TPS) increase chemical diversity by conversion of precursors into hydrocarbons through complex lysis and allylic diphosphate ester bond cleavage (Christianson, 2017). The resulting compounds are structurally related forming variably sized and species-specific gene families (Pichersky and Raguso, 2018).

Phylogenetic analysis of TPSs results in the clustering of genes into seven subfamilies based on sequence homology, gene architecture and functional studies in various plants (Chen et al., 2011). Generally, sesquiterpene synthases (C15) fall primarily into TPS-a, monoterpene synthases (C10) in TPS-b and TPS-g, and diterpene synthases (C20) in TPS-c and TPS-e/f whereas TPS-d and TPS-h are specific to gymnosperms and lycophytes respectively. This vast chemical diversity results in species-specific complex mixtures of terpenes with broadly varying biological activity. Terpenoids mediate interactions between plants and their environment and are critical in coordinating responses to (a)biotic stress (Pichersky and Raguso, 2018). Induced volatile terpene blends composed mainly of lower molecular weight monoterpenes and sesquiterpenes have additional functions in inter and intra-plant communication (He et al., 2022).

Legumes are important crops providing a significant portion of protein in human and animal diets and play an important role in agricultural sustainability. Early investigations in *M. truncatula*, have demonstrated that differential terpene-enriched volatile blends are induced in response to herbivores (Leitner et al., 2005; Arimura et al., 2008). Subsequent studies identified biosynthetic genes underlying these observed metabolites summarized in **Figure 1** (Gomez et al., 2005; Arimura et al., 2008; Navia-Giné et al., 2009; Garms et al., 2010). *Mt*TPS1 produces a sesquiterpene, (*E*)-β caryophyllene, which in maize is instrumental in indirect defense by attracting parasitoids of herbivores and entomopathogenic nematodes (Rasmann et al., 2005; Degenhardt et al., 2009). *Mt*TPS3 forms nerolidol and geranyllinalool common to many plants with defense capabilities. In *M. truncatula*, *Mt*TPS4 which encodes for a monoterpene β-ocimene, was upregulated in response to methyl jasmonate and feeding insects (Navia-Giné et al., 2009). β-ocimene has a priming effect that activates a defense response in Chinese cabbage and cucumber (Navia-Giné et al., 2009; Kang et al., 2018; He et al., 2022). *Mt*TPS5 is a multiproduct TPS, producing up to twenty-seven different terpene products (Garms et al., 2010). More recently, Yadav et al. (2019) showed that *Mt*TPS10, which catalyzes the formation of a sesquiterpene alcohol, himachalol, was upregulated in the root upon infection by an oomycete pathogen, *Aphanomyces euteiches* (Yadav et al., 2019). The combined literature illustrates that *M. truncatula* produces a blend of terpene-based compounds upon interactions with biotic stressors as part of its chemical response strategy.

Soybean terpene synthases *Gm*TPS3, *Gm*TPS18 and *Gm*TPS21 have been characterized and shown to produce β-ocimene, geraniol and α-farnesene respectively, each of which have demonstrated protective roles in plants (Liu et al., 2014; Lin et al., 2017; Han et al., 2023). For example, *Gm*TPS21 (which encodes for α-farnesene) provided resistance against soybean cyst nematode belowground and aphids aboveground (Lin et al., 2017; Constantino et al., 2021). In cowpea, terpenes were identified as part of the volatiles produced in response to herbivory whereas biochemical characterization of the genes responsible for floral scent was carried out in sweet pea (Bao et al., 2020; Steinbrenner et al., 2022).

In grasses, such as maize, rice and wheat, terpenes play an important role in the interactions of plants with their environment (Rasmann et al., 2005; Schnee et al., 2006; Schmelz et al., 2014). Although terpenes are widespread there still exist distinct chemotypes in species with new chemistry and biological roles which are still being uncovered (Pichersky and Raguso, 2018; Ding et al., 2020; Murphy and Zerbe, 2020; Zhang et al., 2021). Comparatively less is known about the composition of the terpenome in legumes.

Here, we define the gene-to-metabolite relationships underlying terpene biosynthesis in *M. truncatula.* Through genome-wide identification of terpene synthases and biochemical characterization of the TPS-a subfamily, we have identified sesquiterpene synthases essential in generating the sesquiterpene-based chemical diversity in *M. truncatula*. Understanding the terpenoid biosynthetic capacity is a foundational step to defining the ecological roles of this important family of compounds.

## Materials and methods

### *Mt*TPS phylogenetic and sequence analysis

Putative terpene synthases (protein models) from the *Medicago truncatula* cv. Jemalong A17 were collated from the Mt4.0v1 genome in Phytozome (https://phytozome-next.jgi.doe.gov) through an annotation-based search for TPSs. The putative TPSs were further manually curated for the presence of catalytic domains namely the Class I site with characteristic DDxxD and secondary NSE/DTE metal binding motif involved in ionization-dependent cyclization. Class II synthases were identified by the DxDD motif involved in protonation-initiated cyclization characteristic of copalyl diphosphate synthases (CPPs) as shown in **Supplementary Figure S1** (Christianson, 2017). For phylogenetic analysis, previously reported and biochemically characterized genes were collated from *S. lycopersicum* ITAG2.4 and *A. thaliana* TAIR10 genomes (Zhou and Pichersky, 2020), aligned with *M. truncatula* TPSs using maximum likelihood analysis with 1000 bootstrap repetitions using CLC software and visualized using iTOL (Letunic and Bork, 2021).

### Combinatorial expression in *Escherichia coli*

Synthetic *Mt*TPSs codon-optimized for expression in *Escherichia coli* were ordered through ThermoFisher and subcloned into pET28b using primers listed in **Supplementary Table S1**. Functional analysis of genes was carried out using a previously described modular metabolic engineering system in *E. coli* enhanced for sesquiterpene production (Kitaoka et al., 2015). Briefly, TPS-a family genes in pET28b were co-transformed with an (*E*, *E*)-FPP synthase from *Zea mays* (*Zm*FPPs) in *E. coli* BL21 DE3-C41 cells (Lucigen). Transformed cultures were grown in 45 mL of Terrific Broth medium to an OD_600_ of ∼0.6 at 37°C Cultures were cooled to 16°C before induction with 1 mM isopropyl-thiogalactopyranoside and supplemented with 1 mM MgCl_2_ and 25 mM sodium pyruvate incubation for 72 hours. Dodecane (20 mL) was added to ensure the capture of volatile compounds. In a second flask, enzyme products were extracted with 50 mL of 100% hexane and concentrated under an N_2_ stream. Samples were resuspended in 1 mL of *n*-hexane for analysis by gas chromatography–mass spectrometry (GC-MS).

### GC-MS analysis

Samples were analyzed on an Agilent 8890B gas chromatograph (GC) coupled to a mass spectrometer (MS) 5977B extractor electron ionization detector at 70 eV. Samples (1 μL) were injected in pulsed spitless mode with the inlet temperature set to 250°C. Separation was achieved on an HP5MS column (30 m, 0.25 mm i.d., 0.25 μm film) using helium as the carrier gas at a flow rate of 1.2 mL/min. The initial oven temperature of 70°C was increased after 1 min to 260°C at a rate of 10°C/min and held for 2 min at 260°C. MS data was collected from 60 to 500 mass-to-charge ratio(m/z). Compounds were identified by comparison of mass spectra and retention times with those of the authentic standards, when available, or with the Wiley, National Institute of Standards and Technology (NIST) mass spectral library.

### Product purification for NMR analysis

To obtain sufficient compound for structural elucidation by NMR, cultures were scaled up to 500 mL batches to a total volume of 5 Liters and expressed using the combinatorial expression system described above (Kitaoka et al., 2015). The resultant products were extracted with an equal volume of hexanes. The organic extract was recovered using a separatory funnel and then dried by rotary evaporation. The resulting residue was passed through a silica column and eluted using a hexane: ethyl acetate gradient. Fractions of interest were further purified by HPLC using an Agilent 1260 series instrument equipped with an autosampler, fraction collector and diode array UV detector over a ZORBAX Eclipse XDB C18 (4.6 x 150 mm, 5 mm) at a 0.5 mL/min flow rate using a water and acetonitrile gradient as the mobile phase. Purified compounds were resuspended in CDCl_3_. Structural analysis was performed using 1D (^‘^1H, 13C^’^) and 2D (TOCSY, ROESYPHPR, HMBCGP, DQFCOSY, and 13CHSQC) NMR experiments. Spectra were acquired on a Bruker Avance 500-MHz TopSpin NMR spectrometer.

### Plant material, growth conditions and elicitors treatment method

Germination of *M. truncatula* cv. Jemalong A17 seeds were carried out by scarification using concentrated anhydrous sulfuric acid for 5 min followed by intensive washing steps with Milli-Q water. Seeds were then placed in water in a 50 ml falcon tube at 4°C for 3 days to break dormancy. Seeds were transferred to moist blotting paper and placed on a petri dish incubated at 26°C. Single seedlings were transferred to pots (9 cm diameter) filled with a perlite and sand mixture (5:1), then grown in a growth chamber with 16 h light/8 h dark for 6 weeks. After 6 weeks of growth, plants were treated by foliar application with solutions containing 1 mM methyl jasmonate (MeJA) in 0.01% Triton-X-100 (Sigma-Aldrich, St Louis, MO, USA) or 1.5 mM salicylic acid solution dissolved in 1% ethanol (SA) (Sigma-Aldrich, St Louis, MO, USA). Plants were treated with 0.01% Triton-X-100 for MeJA control and 1% ethanol for SA. For each plant, 20 ml solution was applied (either MeJA or SA or controls) and at least three biological replicates were conducted for each treatment. The leaf and root tissues were separately collected in time courses from 0h, 2 h, 6 h, 12 h, 24 h, 48 h, 72 h and 96 h and immediately frozen in liquid nitrogen and stored at -80°C for further RNA extraction.

### RNA extraction and cDNA synthesis from root and leaf tissues

Sampling was performed on three biological replicates for both elicitors and controls. The root and leaf tissues were separately ground in liquid nitrogen and 100 mg powder was used for total RNA isolation with an RNeasy Plant Mini Kit (Qiagen), and RNase-Free DNase to remove genomic DNA following the manufacturer’s protocol. RNA purity and yield were checked using a NanoDrop 8000 Spectrophotometer (ThermoFisher Scientific), while RNA integrity and quantification were evaluated with an Agilent 2100 bioanalyzer (Agilent Technologies). First-strand cDNA was synthesized using a Superscript IV Reverse transcriptase kit (ThermoFisher Scientific, USA).

### Quantitative real time-PCR

Primers were designed using coding sequences from the genome of *M. truncatula* (Mt4.0v1) in Phytozome13 (https://phytozome-next.jgi.doe.gov/) (**Supplementary Table S2**). A quantitative real-time PCR system was carried out using SYBR green with ROX (pre-mixed) as an internal loading standard performed on an Eppendorf Mastercycler. The reaction mixture was 10 µl and comprised of 5 µl of 2× SYBR™ Green qPCR Master Mix (Thermofisher Scientific, USA), 100 nM primers (Thermo Fisher Scientific) and 0.5 µl of (1:10 dilution) cDNA. PCR protocol was followed to initiate polymerase activation: 10 min at 95°C; 40 cycles of 30 s at 95°C, 60 s at 55°C and 30 s at 72°C. Each run of qPCR was followed by a melting curve analysis from 55 to 95 °C. PCR conditions were determined by comparing the threshold values of the RT product (cycle threshold (Ct) value). Relative RNA levels were calibrated and normalized with the level of actin and histone-3 reference genes based on the 2^–ΔΔCt^ method (Rey et al., 2016; Zhang et al., 2020). For each gene, three technical replicates were run for three biological samples. qPCR data were expressed as a fold change with respect to the equivalent time-point in the control groups of MeJA (in 0.01% Triton-X-100 and SA (in 1% ethanol).

## Results

### Genome-wide analysis of *Mt*TPSs

**Figure 2:**
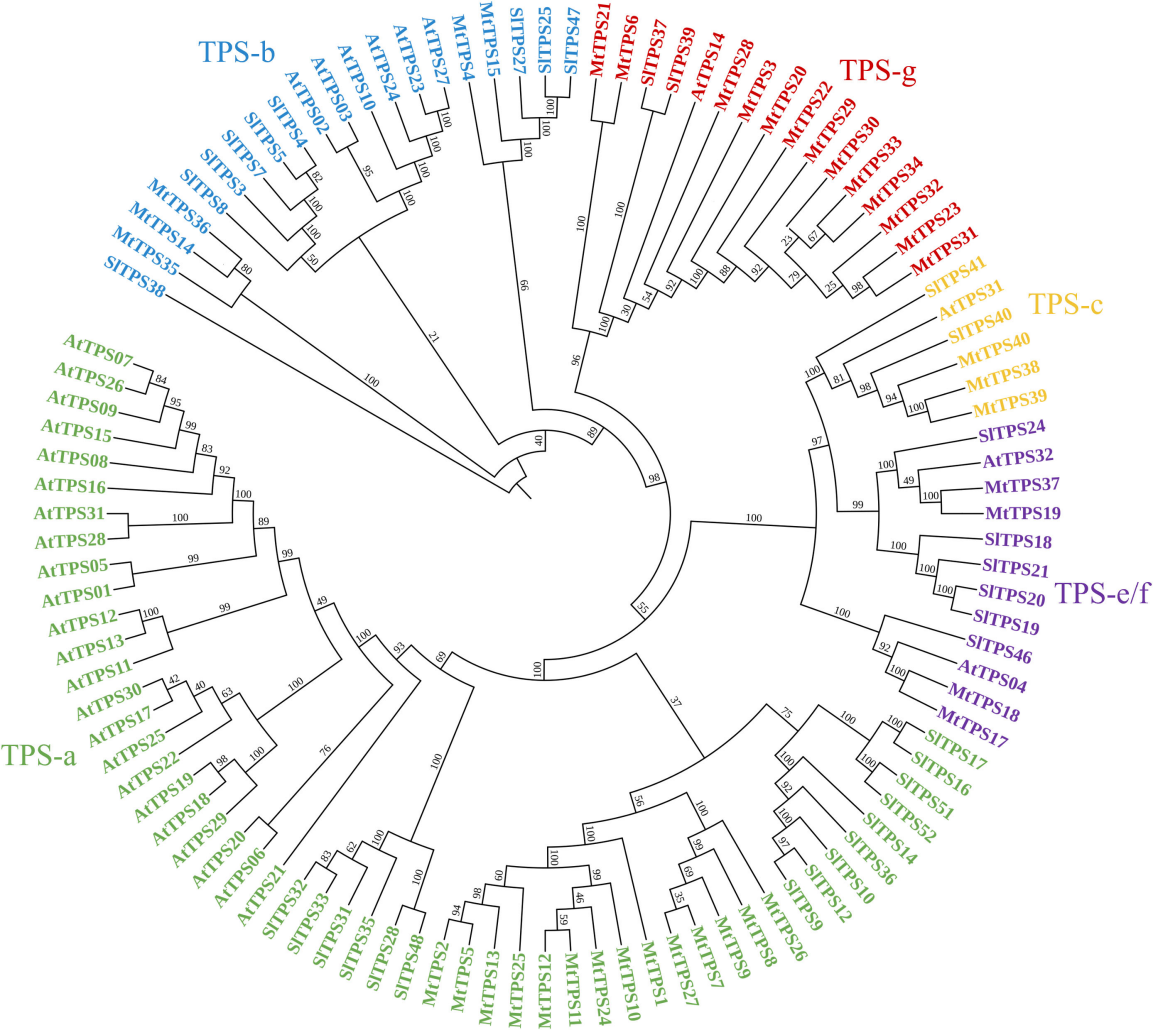
Phylogeny-based analysis of terpene synthases in *Medicago truncatula, Arabidopsis thaliana*, and *Solanum lycopersicum*. The sequences were aligned using maximum likelihood analysis with 1000 bootstrap repetitions using CLC software and visualized using iTOLTPS subfamilies are grouped as follows: TPS- a (green), monoterpenes in TPS-b (blue) and TPS-g (red), and diterpenes in TPS-c (orange) and TPS-e/f (purple).

We assessed for the genetic potential of *M. truncatula* cv. Jemalong A17 to produce terpenes using the Mt4.0v1 genome in Phytozome. We identified 54 putative *Mt*TPS gene models using BLAST and subsequent analysis of characteristic motifs (**Supplementary Table S3** and **Supplementary Figure S1**). Further curation through sequence alignments confirmed thirty- nine full-length genes. Twenty-two of the reported fifty-four potential TPSs in *M. truncatula* were previously assigned numbers by Parker et al., 2014 (*Mt*TPS16 is no longer classified as a putative terpene synthase) (Parker et al., 2014). **Supplementary Table S3** is a summary of the previous and newly assigned TPS numbers. A majority of the genes had the Class I DDxxD and secondary NSE/DTE metal binding motif involved in ionization-dependent cyclization (Christianson, 2017). Three Class II synthases were identified by DxDD motifs involved in protonation-initiated cyclization characteristic of copalyl diphosphate synthases (CPPs) involved in diterpene scaffold formation. Phylogenetic analysis of the terpene synthases from *M. truncatula* and the comprehensively biochemically characterized TPS collections of *A. thaliana* and *S. lycopersicum* shows plant species clustering more than enzymes with similar functions (**Figure 2**) (Chen et al., 2011; Falara et al., 2011; Parker et al., 2014; Zhou and Pichersky, 2020). The genes are distributed into the different clades with fourteen members in TPS-a; five members in TPS-b; three members in TPS-c and four members in TPS e/f. Interestingly there is an expanded TPS-g (thirteen members) subfamily as compared to *A. thaliana* and *S. lycopersicum*.

The biochemical analysis described here focuses on the TPS-a subfamily only, guided by previous studies that showed sesquiterpene compounds are a predominant component of volatile collections of *M. truncatula* in response to herbivory (Leitner et al., 2005; Arimura et al., 2008). In addition, subsequent biochemical characterization assigned metabolites for three of fourteen sesquiterpene synthases - namely *Mt*TPS1, 5, and 10 - which are in the TPS-a subfamily (**Figure 1**) (Gomez et al., 2005; Arimura et al., 2008; Garms et al., 2010). MtTPS2 showed increased gene expression upon elicitation and was putatively described as germacrene-D synthase based on phylogenetic analysis but there was no corresponding biochemical confirmation (Gomez et al., 2005). **Figure 2** shows little overlap of the members of TPS-a clade amongst plants as they tend to group by species. In *S. lycopersicum* the fifteen TPSs encode for sesquiterpenes whereas *Arabidopsis* TPS-a members are not limited to sesquiterpene biosynthetic genes (Zhou and Pichersky, 2020). Combined, these observations guided our investigations to determine the functional landscape of TPS-a subfamily members in *M. truncatula* through heterologous expression.

### Biochemical analysis of TPS-a subfamily

**Figure 3:**
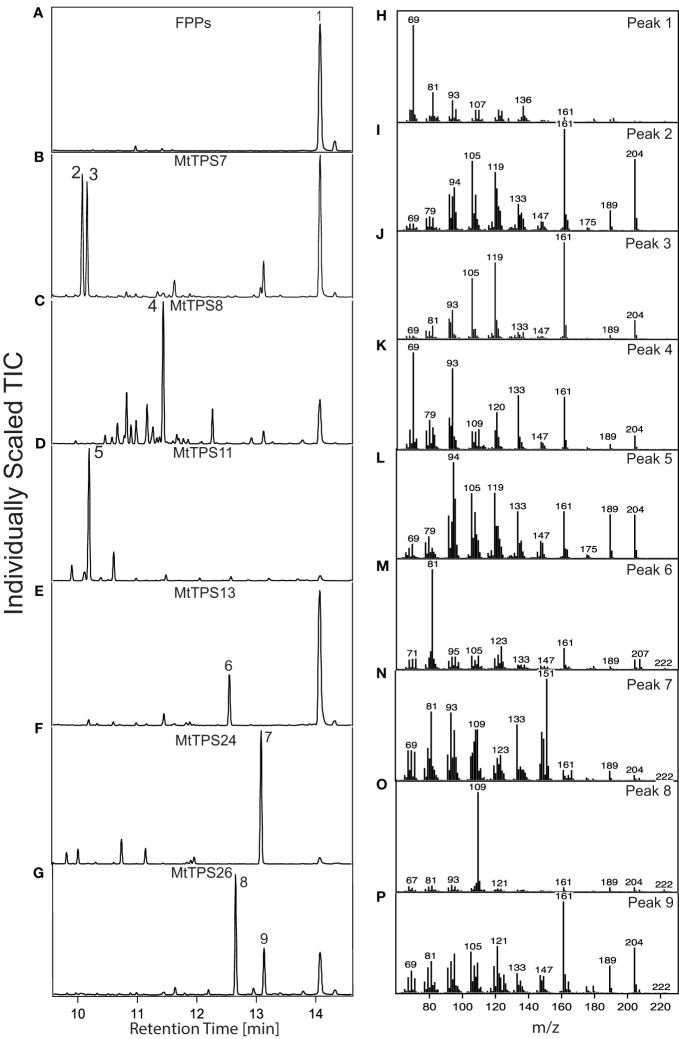
Functional of the TPS-a subfamily in *Medicago truncatula.* Total ion chromatograms of products resulting from combinatorial expression in *E. coli* of (*E*, *E*)-FPP synthase and corresponding genes from the TPS-a subfamily. The chromatograms focus on the main products. Detailed analysis of the smaller peaks is depicted in **Supplementary Figures S3**-**8**. Compound identification was through similarity to library matches (NIST, Wiley) for B-E and NMR verification for G. **(A)** pET28b containing FPP synthase produces **(H)** dephosphorylated FPP (farnesol) (1) **(B)** *Mt*TPS7 produces **(I, J)** cyclosativene (2), α-copaene (3). **(C)** *Mt*TPS8 produces **(K)** farnesene-like product (4). **(D)** *Mt*TPS11 produces **(L)** longicyclene (5). **(E)** *Mt*TPS13 makes a **(M)** germacrene-D-4-ol product (6), **(F)** *Mt*TPS24 makes a **(N)** unknown product (7) and **(G)** *Mt*TPS26 makes a **(O, P)** eudesm4(14)-en-6β-ol (8) and a guai-6-en-10β-ol (9).

To correlate metabolites produced to the identified biosynthetic genes, we assessed for TPS activity and resultant metabolite formation using a modular metabolic engineering system in *E. coli* (Kitaoka et al., 2015). Briefly, heterologous expression was performed through co-expression of an (*E*, *E*)-FPP synthase with uncharacterized full-length TPS-a subfamily genes (*Mt*TPS2, 7, 8, 9, 11, 12, 13, 24, 25, 26 and 27). Metabolite production was analyzed using GC-MS (**Figure 3**). Compounds were compared to authentic standards when available, the NIST library database, or verified through *de novo* structural elucidation by NMR.

Analysis of resultant metabolites produced by TPS-a subfamily members reveals the production of an assortment of sesquiterpenes. **Figure 3** highlights the most abundant products formed by TPSs and products in smaller quantities are described in the supplementary information (**Suppplementary Figures S3**-**S8**). *Mt*TPS7 is a multiproduct enzyme that produces α-copaene (2), cyclosativene (3) and a hydroxylated compound which matches a compound produced by *Mt*TPS26 (**Figure 3B**; **Supplementary Figure S3**). *Mt*TPS8 is a multiproduct synthase that produces farnesene (4) as the major compound and exhibits relatively significant turnover to amorphadiene, bergamotene and nerolidol, and production of up to fourteen products in smaller amounts (**Figure 3C**; **Supplementary Figure S4**), *Mt*TPS11, produces longicyclene (5) as the main product and a smaller amount of longifolene and longipinene (**Figure 3D**; **Supplementary Figure S5**).

Although terpene synthases more commonly form hydrocarbons, the addition of water before terminating deprotonation generates hydroxylated products (Christianson, 2017). *M. truncatula* produces a set of TPSs capable of producing such hydroxylated compounds as supported by a molecular weight of 222. *Mt*TPS13 produces germacrene-D-4-ol (6) as compared to NIST (**Figure 3E**; **Supplementary Figure S6**). The genes, *Mt*TPS24 and *Mt*TPS26 are present in the *Mt*4.0v1 genome and not identified in earlier genomes (Parker et al., 2014). *Mt*TPS24 produces a compound with a distinct base peak at 151 (7) (**Figure 3F**; **Supplementary Figure S7**). However, we were unable to collect NMR data as the compound aggregates in tested deuterated solvents (chloroform, methanol and DMSO) and no further analysis could be performed. Upon expression, *Mt*TPS26 produces two predominant compounds that required structural verification by NMR due to the unavailability of authentic standards. Structural elucidation by NMR and comparison to literature values showed that compound 8 was eudesm4(14)-en-6β-ol (**Figure 3G**) with chemical shift data closely matching previously reported data for 10-*epi*-junenol except for the hydroxyl in a 6β-position in 8 (**Supplementary Table S5**) (F. et al., 1976). Compound 9 was confirmed to be guai-6-en-10β-ol, matching previously reported chemical shifts as shown in **Supplementary Table S6** (Lago et al., 2000). No activity was detected with *Mt*TPS2, 9, 12, 25 and 27 upon co-expression with (*E*, *E*)-FPP synthase using the combinatorial expression system.

The ability of TPSs to make several products is not uncommon, for example, *Mt*TPS5 was demonstrated to produce up to twenty-seven products (Garms et al., 2010; Vattekkatte et al., 2017). Here, *Mt*TPS8 is the most promiscuous forming up to twenty compounds (**Supplementary Figure S4**). Multiple products produced in smaller quantities are highlighted for all the genes (**Supplementary Figures S3**-**S8**). Similarly, because of the related mechanism of terpene synthases, it is common to observe that some genes will also produce the same compounds in varying amounts *in vitro*. This is observed for *Mt*TPS7 and *Mt*TPS26 which both produce 9; a similar case is noted for *Mt*TPS11 which produces longicyclene (5) as the main product, but profiles of *Mt*TPS5 & 10 also produce longicyclene in small amounts (Garms et al., 2010; Yadav et al., 2019).

**Figure 4:**
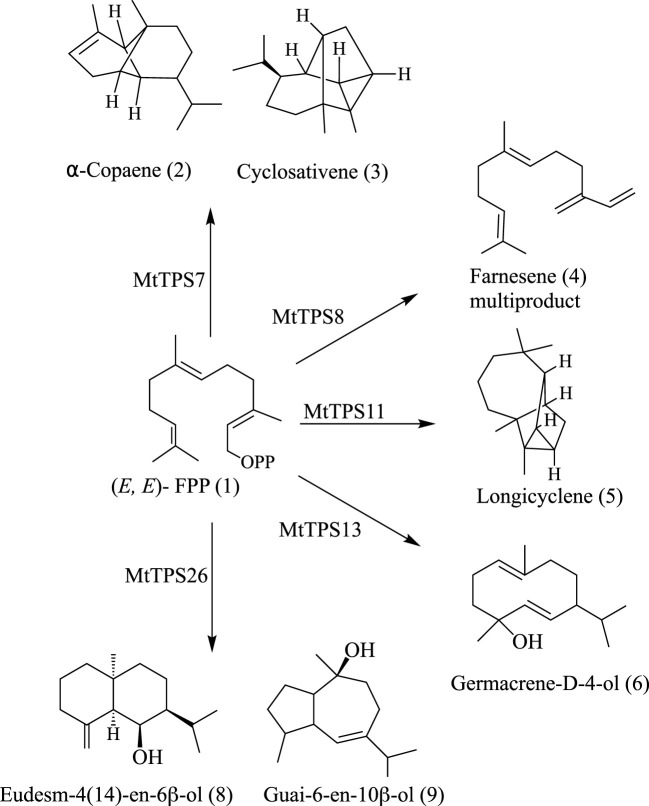
Summary of the main products from biochemical characterization of TPS-a subfamily members in *M. truncatula*.

In summary, this biochemical analysis defines gene-to-metabolite relationships in the TPS-a subfamily of *M. truncatula*. We defined the genetic basis for the biosynthesis of farnesene, nerolidol, cyclosativene, copaene and longicyclene, enabling the association of genes responsible for the biosynthesis of several compounds observed in terpene metabolite response to herbivore feeding (Leitner et al., 2005) and identified a set of sesquiterpene alcohols (**Figure 4**).

### Gene expression analysis of TPS-a members in response to treatment with methyl jasmonate and salicylic acid

**Figure 5:**
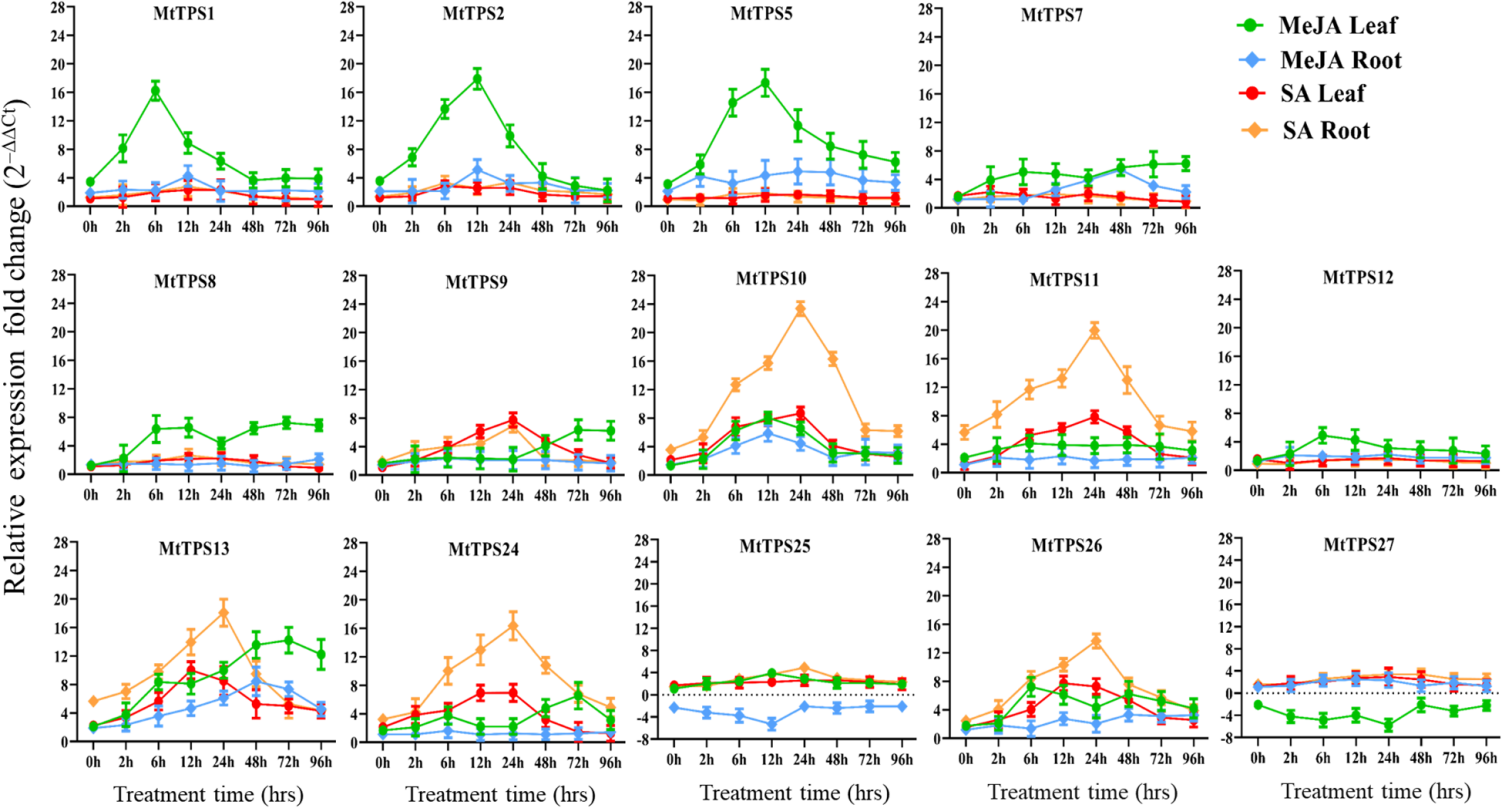
Expression profile of TPS-a terpene synthase genes in leaf and root tissues in response to exogenous application of MeJA and SA in a time course exposure by qRT-PCR. Plants were exposed for 0h, 2 h, 6 h, 12 h, 24 h, 48 h, 72 h and 96 h after foliar application of MeJA (1 mM) or SA (1.5 mM). The data are expressed as the relative level of expression of fold change relative to controls (0.01% Triton-100 for MeJA, or 1% ethanol for SA control) plants, based on the 2^−ΔΔct^ method. Error bars represent the mean ± standard deviation (SD) levels of the relative abundance of three biological replicates.

Gene expression of the TPS-a subfamily members in response to elicitors was carried out through exogenous application of phytohormones MeJA and SA and analysis by qPCR (**Figure 5**). Overall, TPS-a genes showed tissue-specific and temporal differences in expression in response to elicitation with MeJA and SA. There was higher expression of TPS-a genes in leaf tissue compared to the root upon elicitation with MeJA. Specifically, our time course evaluations revealed that MeJA application resulted in increased expression (16-20 fold) of *Mt*TPS1 after hours of exposure in leaves whereas expression of *Mt*TPS2 and *Mt*TPS5 was highest after 12 hours. A 4-8 fold increase in expression was observed amongst the *Mt*TPS8, *Mt*TPS10, *Mt*TPS13 and *Mt*TPS26 genes in leaf tissues after 6 and 12 hours of post-treatment of MeJA. *Mt*TPS11, 12, 24, 25 & 27 have low expression in both the leaves and roots. There was a consistently low expression for all the TPS-a members in roots under MeJA elicitation, with MtTPS25 & 27 showing down-regulation.

In contrast, SA induced gene expression occurred mainly in roots. The genes *Mt*TPS10 and *Mt*TPS11 showed the highest expression at 24 hours compared to control with a 20-fold increase whereas a 16-fold increase in expression was observed for genes *Mt*TPS13, 24 & 26 (**Figure 5**). However, comparatively lower expression was observed in both leaf and root tissue for the remaining eight genes (*Mt*TPS1, 2, 5, 7, 8, 12, 25 & 27). Overall, the TPS-a genes exhibited differential expression to the phytohormones where MeJA largely affected the expression of TPS genes in leaf tissue and SA in root tissue upon foliar treatment.

### Expression patterns of TPS in different tissues and under (a)biotic stress

We used a publicly available expression data set of *M. truncatula* from the Noble Research Institute legacy Gene Atlas V3 to surve*y* the effect of (a)biotic stress on the TPS-a subfamily (Benedito et al., 2008; Carrere et al., 2021). Mining of the reference data set, which is a collation of gene expression in different tissues, shows that terpene synthases *Mt*TPS1, 2 & 5 which produce volatile compounds exhibited increased expression in the shoots and leaves. Transcript levels of the *Mt*TPS13 were increased during root and nodule development, AM symbiosis, and fungal colonization (**Supplementary Figure S23A**).

Evaluation of the *TPS*-a subfamily genes response to abiotic stress highlights increased transcript abundance of *Mt*TPS1, 2, 5 and *Mt*TPS13 under limited nitrogen (N_2_), ammonium (NH_4_), nitrate (NO_3_), drought, and salt stress in shoot and root tissues (**Supplementary Figure S23B**). An assessment of varied biotic stressors showed that *Mt*TPS1 and 2 had increased expression in leaf tissue after aphid infestation whereas *Mt*TPS13, *Mt*TPS24 and *Mt*TPS25 displayed increased transcript abundance in root tissues under pathogenic fungi, *Rhizoctonia solani* and *Fusarium oxysporum* (**Supplementary Figure S23C**). Notably, datasets obtained from Gene Atlas do not capture the expression of six terpene synthases (*Mt*TPS7,8, 9, 25, 26 & 27). Combined this data supports the variable expression of select terpene synthases dependent on tissue, time and stressor applied.

## Discussion

### Biochemical analysis reveals functional divergence of TPS-a subfamily

The catalytic mechanism of sesquiterpenes proceeds via a succession of carbocationic intermediates initiated by dephosphorylation and subsequent deprotonation or water capture (Christianson, 2017). The metabolites of the encoded enzyme of *Mt*TPS1, *Mt*TPS7 and *Mt*TPS8 have characteristic profiles to previously described enzymes, for example, *Mt*TPS8 produces (*E*)-β-farnesene with bergamotene similar to what is observed for *Zm*TPS10 (Schnee et al., 2002; Rasmann et al., 2005; Schnee et al., 2006). Notably, *Mt*TPS11 produces longicyclene, a compound commonly found in pine trees (Martin et al., 2004). Previous studies have identified a longifolene synthase with smaller amounts of longicyclene and longipinene - here the enzyme *Mt*TPS11 forms longicyclene as the major product. Olefins (compounds 2-5) produced by *M. truncatula* are characteristic in volatile blends of several plants upon herbivore feeding and associated interactions (Schnee et al., 2002; Schnee et al., 2006). Here, some of the TPS-a genes quenched the reaction through water capture resulting in sesquiterpene alcohols (compounds 6-9) that have been identified in other plants, but little is known of their biological activity. Our biochemical analysis combined with the previously characterized genes establishes the use of terpenes common to plants generating a species-specific sesquiterpene blend.

A summary of the known gene-to-metabolite relationships in the TPS-a subfamily of *M. truncatula* shows functional divergence from the TPS profiles of the two comprehensively biochemically characterized eudicots (**Figure 4**). Previously characterized genes from *M. truncatula* mainly fall in the TPS-a clade (**Figures 1** & **2**). Comparison of the currently defined *M. truncatula* TPS-a subfamily members compared to those of *Arabidopsis* and tomato show a difference in metabolite profiles. In tomato, the fifteen TPSs encode for sesquiterpenes whereas the product outcome of the TPS-a subfamily in *A. thaliana* is not limited to sesquiterpenes (**Supplementary Table S4**) (Zhou and Pichersky, 2020). Biochemical analysis here revealed that a subset of the metabolites synthesized by *M. truncatula* sesquiterpene synthases was specific to the species, revealing functional divergence.

### Differential expression of TPS-a subfamily in response to phytohormone elicitation

In this study, we elicited TPS expression by exogenous application of phytohormones. The genes that produce volatile compounds had increased expression on the early onset of foliar application of MeJA (**Figures 1**, **3**). Genes corresponding to the production of volatile compounds β-caryophyllene (MtTPS1), putative germacrene-D (*Mt*TPS2) and *Mt*TPS5 a multiproduct synthase with the demonstrated antiherbivore response were upregulated by 16 to 20-fold (**Figure 5**). These observations correlate in part to a study by Leitner et al. (2005), which showed that herbivore interactions and artificial wounding induced the emission of up to 23 compounds (Leitner et al., 2005; Arimura et al., 2008). This observation is supported by data from gene atlas that also showed increased expression of the volatile terpenes predominantly in the leaves under various stresses (**Supplementary Figure S23**). However, ten out of fourteen TPS-a subfamily members exhibited moderate upregulation by 4-6-fold using MeJA.

In contrast, elicitation using SA increased the transcript abundance in root tissue of select genes; *Mt*TPS10, *Mt*TPS11, *Mt*TPS13, *Mt*TPS24 and *Mt*TPS26 (**Figure 5**). In general, SA mediates response to biotrophic and hemibiotrophic pathogens and triggers systemic acquired resistance (Anand et al., 2008). Yadav et al. (2019) demonstrated that MtTPS10, which produces himalachol, reduced susceptibility to the oomycete pathogen *Aphanomyces euteiches* (Yadav et al., 2019). The terpene scaffolds may be elaborated by downstream enzymes resulting in further oxygenated non-volatile sesquiterpenoids. The resultant compounds may have various bioactivity similar to previously identified antifungals and insect deterrents such as germacrene-A based costunolides in lettuce; costic acids derived from selinene and β-macrocarpene based zealexins produced in maize under pathogen attack (Nguyen et al., 2010; Ding et al., 2017; Ding et al., 2019).

In *conclusion*, the results reported here establish gene-metabolite relationships of the TPS-a subfamily that encodes for sesquiterpene synthases in *M. truncatula.* Biochemical analysis reveals a blend of sesquiterpenes that highlight the evolution of species-specific chemotypes. Gene expression analysis shows a temporal tissue-specific and stress induced variation. While determining the function of these compounds is beyond the scope of this work, our study further defines the sesquiterpene biosynthetic capacity and provides a foundation to understand the biological roles of the compounds.

## Data availability statement

The original contributions presented in the study are included in the article/**Supplementary Material**. Further inquiries can be directed to the corresponding author.

## Author contributions

HH: Investigation, Methodology, Writing – original draft, Writing – review & editing. MI: Investigation, Writing – original draft. GW: Formal analysis, Writing – review & editing. SM: Conceptualization, Formal analysis, Funding acquisition, Investigation, Methodology, Project administration, Resources, Supervision, Writing – original draft, Writing – review & editing.
