# Supplementary material for: Biochemical analysis of the TPS-a subfamily in Medicago truncatula
Source: Front Plant Sci. 2024 Feb 15;15:1349009. doi: 10.3389/fpls.2024.1349009 (PMC10902008; doi:10.3389/fpls.2024.1349009)
Supplement: Supplementary file 1 [file DataSheet_1.pdf]

Supplementary Material

Title: Biochemical analysis of the TPS-a subfamily in *Medicago truncatula*

Hannah Hendrickson[1], Monirul Islam[2], Ghislain Fotso Wabo[3] and Sibongile Mafu[1, 2*]

*Corresponding Author: smafu@umass.edu

CONTENT

Table of Contents 1
Table S1: Primers for subcloning *Mt*TPSs into expression vectors 3
Table S2: Primers for qPCR analysis 4

Table S3: Complete list of putative *Mt*TPSs collated from the Phytozome Database - *Medicago truncatula* (Mt4.0v1). 5

Figure S1. Characteristic terpene motifs organized by clade. 7

GC-MS data for smaller peaks
Figure S2: Mass spectra comparison to NIST (Wiley). 8
Figure S3: GC-MS of minor compounds produced by MtTPS7. 9
Figure S4: GC-MS of minor compounds produced by MtTPS8. 11
Figure S5: GC-MS of minor compounds produced by MtTPS11. 13
Figure S6: GC-MS of minor compounds produced by MtTPS13. 14
Figure S7: GC-MS of minor compounds produced by MtTPS24. 15
Figure S8: GC-MS of minor compounds produced by MtTPS26. 16

Table S4: Summary of metabolites produced by TPS-a family members in Arabidopsis, *S. lycopersicum* and *M. truncatula* 17

NMR Data
Compound 8
Table S5: ^{13}C and ^{1}H-NMR spectroscopic data of compound **8.** 19
Figure S9: Numbering and main HMBC correlations of compound **8.** 19
Figure S10a: ^{1}H-NMR spectrum ($CDCl_3$, 500 MHz) of compound **8** (full spectrum). 20
Figure S10b: ^{1}H-NMR spectrum ($CDCl_3$, 500 MHz) of compound **8** (upfield). 21
Figure S11: ^{13}C-NMR spectrum ($CDCl_3$, 125 MHz) of compound **8** (full spectrum). 22
Figure S12: ^{1}H-^{1}H COSY spectrum of compound **8** (full spectrum). 23
Figure S13: HMQC spectrum of compound **8** (full spectrum). 24
Figure S14a: HMBC spectrum of compound **8** (full spectrum). 25
Figure S14b: HMBC spectrum of compound **8** (upfield). 26
Figure S15: ^{1}H-^{1}H ROESY spectrum of compound **8** (full spectrum). 27

Compound 9

Table S6: ^{13}C and ^{1}H-NMR spectroscopic data of compound **9.**	28
Figure S16: Numbering and main HMBC correlations of compound **9.**	28
Figure S17a: ^{1}H-NMR spectrum ($CDCl_3$, 500 MHz) of compound **9** (full spectrum).	29
Figure S17b: ^{1}H-NMR spectrum ($CDCl_3$, 500 MHz) of compound **9** (upfield).	30
Figure S18: ^{13}C-NMR spectrum ($CDCl_3$, 125 MHz) of compound **9** (full spectrum).	31
Figure S19a: ^{1}H-^{1}H COSY spectrum of compound **9** (full spectrum).	32
Figure S19b: ^{1}H-^{1}H COSY spectrum of compound **9** (upfield).	33
Figure S20a: HMQC spectrum of compound **9** (full spectrum).	34
Figure S20b: HMQC spectrum of compound **9** (upfield).	35
Figure S21a: HMBC spectrum of compound **9** (full spectrum).	36
Figure S21b: HMBC spectrum of compound **9** (upfield).	37
Figure S21c: HMBC spectrum of compound 9 (downfield).	38
Figure S22a: ^{1}H-^{1}H ROESY spectrum of compound **9** (full spectrum).	39
Figure S22b: ^{1}H-^{1}H ROESY spectrum of compound **9** (upfield).	40

Gene expression data

Figure S23: Gene Atlas data of terpene synthase expression from TPS-a subfamily in *M. truncatula*.	41

***Note**: The Supplementary Information order of Tables and Figures closely follows the order mentioned in the manuscript.*

Table S1. Primers for subcloning of MtTPSs into expression vectors

MtTPS	Forward (F) and Reverse (R) Primers	
sMedtr2g081980 (*MtTPS7*)	F	AGCAAATGGGTCGCGGATCCATGGAAAGCCTGGCAGCACC
	R	GGTGGTGGTGGTGCTCGAGTTCTGACAGTTCAATGCTAA
sMedtr2g082010 (*MtTPS8*)	F	AGCAAATGGGTCGCGGATCCATGGAATACAGCCAGACCAA
	R	GGTGGTGGTGGTGCTCGAGTTTATTCACACTGTTCAATGC
sMedtr2g082060 (*MtTPS9*)	F	AGCAAATGGGTCGCGGATCCATGAGCCTGGCACCGGCAAC
	R	GGTGGTGGTGGTGCTCGAGTTTACTTGCACTGTTCAATAC
sMedtr5g073260 (*MtTPS11*)	F	AGCAAATGGGTCGCGGATCCATGAGCAGCATTGCAAATAG
	R	GGTGGTGGTGGTGCTCGAGTTTACAGCGGCACCGGTTCAA
sMedtr5g094620 (*MtTPS12*)	F	AGCAAATGGGTCGCGGATCCATGAGCAGCCTGAGCAGTCT
	R	GGTGGTGGTGGTGCTCGAGTTTAGATCGGAACCGGATCAA
sMedtr6g008560 (*MtTPS13*)	F	AGCAAATGGGTCGCGGATCCATGAGCCATGCAGCATGTTT
	R	GGTGGTGGTGGTGCTCGAGTTTAGATCGGAACCGGATCAA
sMedtr3g464190 (*MtTPS24*)	F	AGCAAATGGGTCGCGGATCCATGAGCAGCGTTGCATGTCTGAATC
	R	GGTGGTGGTGGTGCTCGAGTTTAAATCAGAACCGGATCAACCAG
sMedtr4g048460 (*MtTPS25*)	F	AGCAAATGGGTCGCGGATCCATGAATAGCAGCGGTGCACT
	R	GGTGGTGGTGGTGCTCGAGTTTAGATCGGAACCGGATCAA
sMedtr8g007515 (*MtTPS26*)	F	AGCAAATGGGTCGCGGATCCATGAGCCTGCCGACCACCAT
	R	GGTGGTGGTGGTGCTCGAGTTTATTCATGCTGTTCGATAC
sMedtr4g019550 (*MtTPS27*)	F	AGCAAATGGGTCGCGGATCCATGAGCCTGGCAGCACCGAG
	R	GGTGGTGGTGGTGCTCGAGTTTATCAGATGCTGATCGGCT

**Primers were designed for synthetic gene sequences optimized for expression in E. coli using BamHI and XhoI restriction sites in the pET28b vector.*

Table S2. Primers for qPCR analysis

MtTPS	Forward (F) and Reverse (R) Primers		Amplicon Length (bp)
Actin *Medtr3g095530*	F	GAATTCACGAGACCACCTACAA	116
	R	GTCAGCAATACCAGGGAACATA	
Histone-3 *Medtr4g097170*	F	CGTGAGATTAGGAAGTATCAGAAGAG	113
	R	CTCTGGAAACGAAGGTCAGTC	
MtTPS1 *Medtr4g081460*	F	GAGCAAGAGATGGTCAAGGAA	105
	R	ACTGAACCATTTAGCCTCAGTAA	
MtTPS2 *Medtr6g039440*	F	CGAGCAAGATCCATCCCATAA	121
	R	GTCCAATTCCTTCCACCATTTG	
MtTPS5 *Medtr5g062230*	F	TGTCGTCTCGGTGTTAGTTATC	125
	R	AGCACAGCGAGAGAGTAAAG	
MtTPS7 *Medtr2g081980*	F	CCTAGGTTAGAGACAAGGTGTTATATT	134
	R	GTGATGCTGCCGATTTCATTT	
MtTPS8 *Medtr2g082010*	F	GCCATGTCCTATGTGCCTAAA	140
	R	CTCTGGATTGCCTGTGTGAATA	
MtTPS9 *Medtr2g082060*	F	GTTGGAGACAAGGTGCTACAT	130
	R	GGTGATACTGCCGAGTTCTTT	
MtTPS10 *Medtr5g073200*	F	GACTGAGGCCAGATGGTTAAA	123
	R	ATGTCTCCCATGCCAATGAA	
MtTPS11 *Medtr5g073260*	F	CACTACCACTCACCTTCAATCC	106
	R	GAGCCGAGGCAAGTTCTTATAG	
MtTPS12 *Medtr5g094620*	F	TATGACTGAGGCTAGATGGTTAAAG	130
	R	AACTGTGTCTCCCATACCAATATAA	
MtTPS13 *Medtr6g008560*	F	CATGGACGGTCTTCCTAACTAC	104
	R	GGGTGTATAGCCTTCCTTCTT1	
MtTPS24 *Medtr3g464190*	F	CTGTTGTACTTTGCAGGCTAATG	120
	R	CAGCTTCCCTAGACACATCATATT	
MtTPS25 *Medtr4g048460*	F	CTGTTGAAGGTTTATGAGGAAATAGAG	124
	R	GCCATCTAGCCTCTGTGATATAAG	
MtTPS26 *Medtr8g007515*	F	GATTGGCACAAGCGTATTTGG	112
	R	GTTGACAGTGGGATGGTAGAAG	
MtTPS27 *Medtr4g019550*	F	CTTCTACACTCTCACTTCAACTTACT	135
	R	GTCATCGGTGAGTCTAGCAATAG	

Table S3. Complete list of putative *Mt*TPSs collated from the Phytozome Database - *Medicago truncatula* (Mt4.0v1).

MtTPS	Phytozome ID	Clade	Peptide Sequence Length	Predicted Class
MtTPS1	*Medtr4g081460*	A	563	C15
MtTPS2	*Medtr6g039440*	A	554	C15
MtTPS3	*Medtr4g092010*	G	574	C10
MtTPS4	*Medtr4g045810*	B	581	C10
MtTPS5	*Medtr5g062230*	A	554	C15
MtTPS6	*Medtr2g089120*	G	568	C10
MtTPS7	*Medtr2g081980*	A	562	C15
MtTPS8	*Medtr2g082010*	A	573	C15
MtTPS9	*Medtr2g082060*	A	558	C15
MtTPS10	*Medtr5g073200*	A	550	C15
MtTPS11	*Medtr5g073260*	A	553	C15
MtTPS12	*Medtr5g094620*	A	538	C15
MtTPS13	*Medtr6g008560*	A	558	C15
MtTPS14	*Medtr2g065450*	B	590	C10
MtTPS15	*Medtr5g010710*	B	554	C10
**MtTPS16*	*Medtr7g011670*	X	X	X
MtTPS17	*Medtr2g012870*	E/F	807	C20
MtTPS18	*Medtr2g012900*	E/F	820	C20
MtTPS19	*Medtr3g058160*	E/F	822	C20
MtTPS20	*Medtr2g010960*	G	521	C10
MtTPS21	*Medtr2g089130*	G	566	C10
MtTPS22	*Medtr3g052120*	G	516	C10
MtTPS23	*Medtr6g064980*	G	520	C10
MtTPS24	*Medtr3g464190*	A	558	C15
MtTPS25	*Medtr4g048460*	A	562	C15
MtTPS26	*Medtr8g007515*	A	561	C15
MtTPS27	*Medtr4g019550*	A	559	C15
MtTPS28	*Medtr4g092020*	G	571	C10
MtTPS29	*Medtr3g063160*	G	291	C10
MtTPS30	*Medtr3g465090*	G	520	C10
MtTPS31	*Medtr6g065040*	G	403	C10
MtTPS32	*Medtr3g063120*	G	496	C10
MtTPS33	*Medtr3g063170*	G	520	C10
MtTPS34	*Medtr3g063140*	G	520	C10
MtTPS35	*Medtr8g057020*	B	590	C10
MtTPS36	*Medtr2g064425*	B	592	C10
MtTPS37	*Medtr2g064295*	E/F	781	C20
MtTPS38	*Medtr7g011663*	C	823	C20
MtTPS39	*Medtr7g011770*	C	718	C20
MtTPS40	*Medtr7g094970*	C	675	C20
Incomplete	*Medtr2g082050*	X	462	X

Incomplete	*Medtr5g094530*	X	189	X
Incomplete	*Medtr7g050990*	X	925	X
Incomplete	*Medtr2g010940*	X	88	X
Incomplete	*Medtr7g057390*	X	170	X
Incomplete	*Medtr7g011690*	X	97	X
Incomplete	*Medtr4g094020*	X	350	X
Incomplete	*Medtr7g010700*	X	87	X
Incomplete	*Medtr5g081730*	X	152	X
Incomplete	*Medtr8g073530*	X	282	X
Incomplete	*Medtr3g034810*	X	106	X
Incomplete	*Medtr1g050430*	X	159	X
Incomplete	*Medtr0534s0030*	X	101	X
Incomplete	*Medtr7g057340*	X	173	X
Incomplete	*Medtr5g030050*	X	314	X

*****MtTPS16 was previously numbered and defined as a putative MtTPSs by Parker et al. in 2014 but it is no longer classified as a putative TPS.*

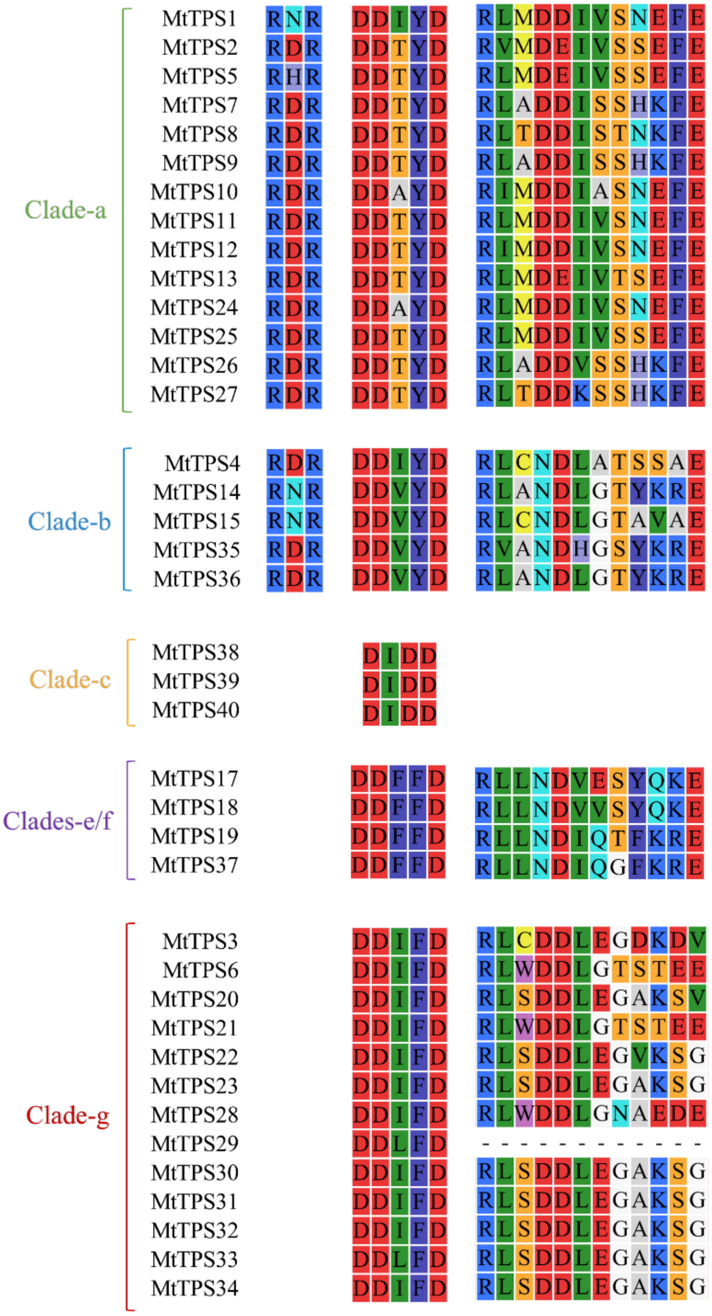

Figure S1. Characteristic terpene motifs organized by clade (TPS-subfamily). Putative sesquiterpene synthases of TPS-a subfamily and putative monoterpene synthases of TPS-b show characteristic RxR, DDxxD, and NSE motifs. Putative diterpene synthases of TPS-c have a DxDD motif characteristic of Class II. e/f show or, respectively. TPS e/f are putative diterpene synthases with a characteristic DDxxD and NSE motifs. Putative monoterpenes in TPS-g show DDxxD and NSE motifs. MtTPS29, while organizing into clade g, does not display the NSE/DTE motif. This may be a putative PTS (Christianson et al. 2017).

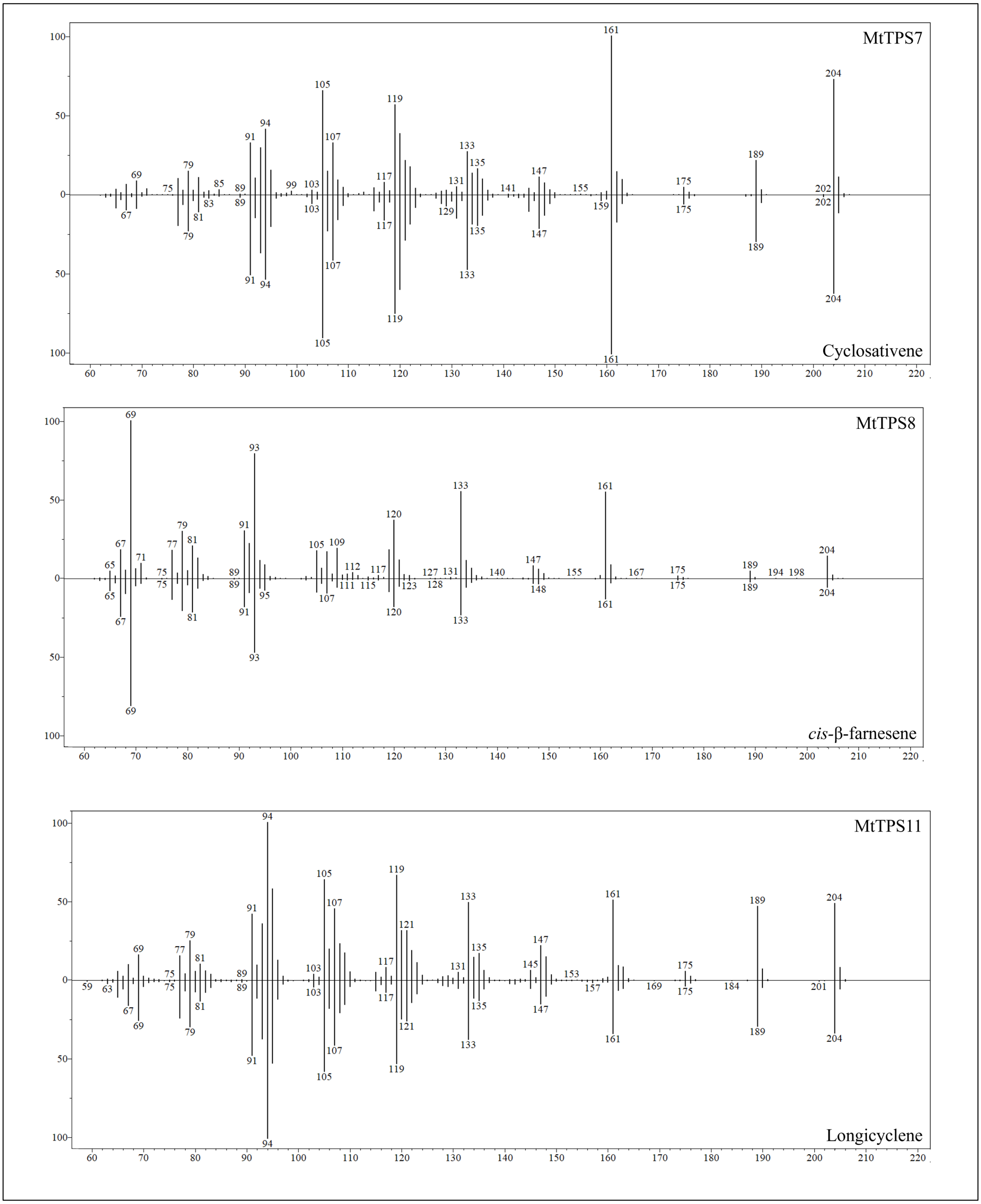
MtTPS7
Cyclosativene
MtTPS8
cis-β-farnesene
MtTPS11
Longicyclene

Figure S2: NIST library comparison

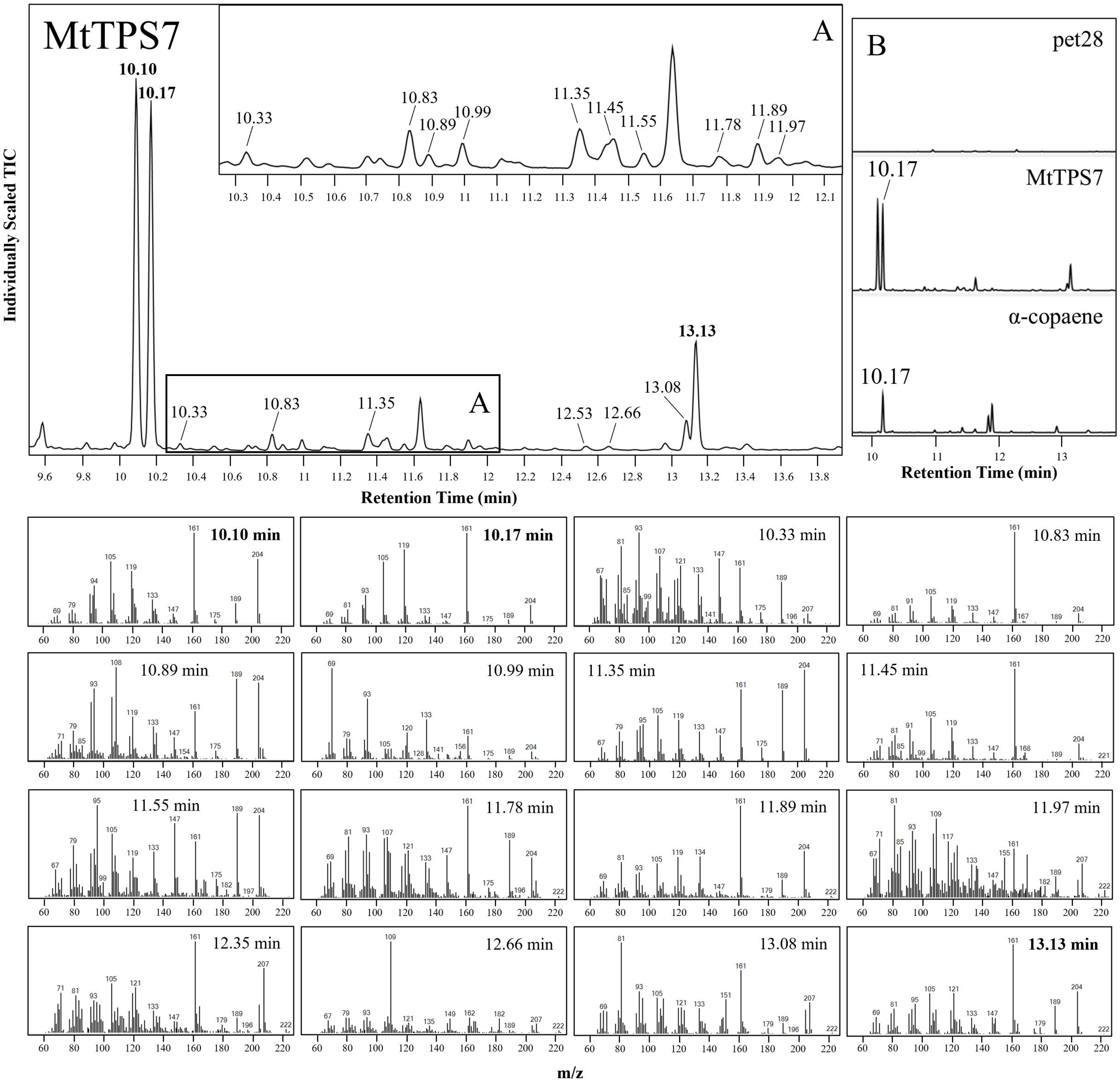

MtTPS7 C15 Peaks							
**10.10 min**	**(+)-cyclosativene**	10.89 min	β-patchoulene	11.55 min	aciphyllene	12.53 min	cubebol
**10.17 min**	**α-copaene**	10.99 min	cis- β-farnesene	11.78 min	(-)-globulol	12.66 min	unknown C15
10.33 min	β-elemene	11.35 min	cyperene	11.89 min	epicubebol	13.08 min	cubebol
10.83 min	β-cubebene	11.45 min	β-cubebene	11.97 min	shyobunol	**13.13 min**	**guai-6-en-10β-ol**

Figure S3: GC-MS of minor compounds produced by MtTPS7.

MtTPS7 is a multiproduct synthase, creating several C15 products in addition to its main products (+)-cyclosativene and α-copaene, which have RTs of and 10.10 and 10.17 min and are highlighted above. MS spectra correspond to labelled peaks (which are labelled according to their retention times) and best identity matches from the NIST database are listed below. Smaller peaks are shown in greater detail in panel A, which corresponds to the similarly labelled area of the GC spectrum.

The *cis*-β-farnesene peak was also seen in the control sample at 11.0 minutes and is not an *MtTPS7* product. Panel **B** shows comparison of the *MtTPS7* α-copaene product to an α-copaene standard.

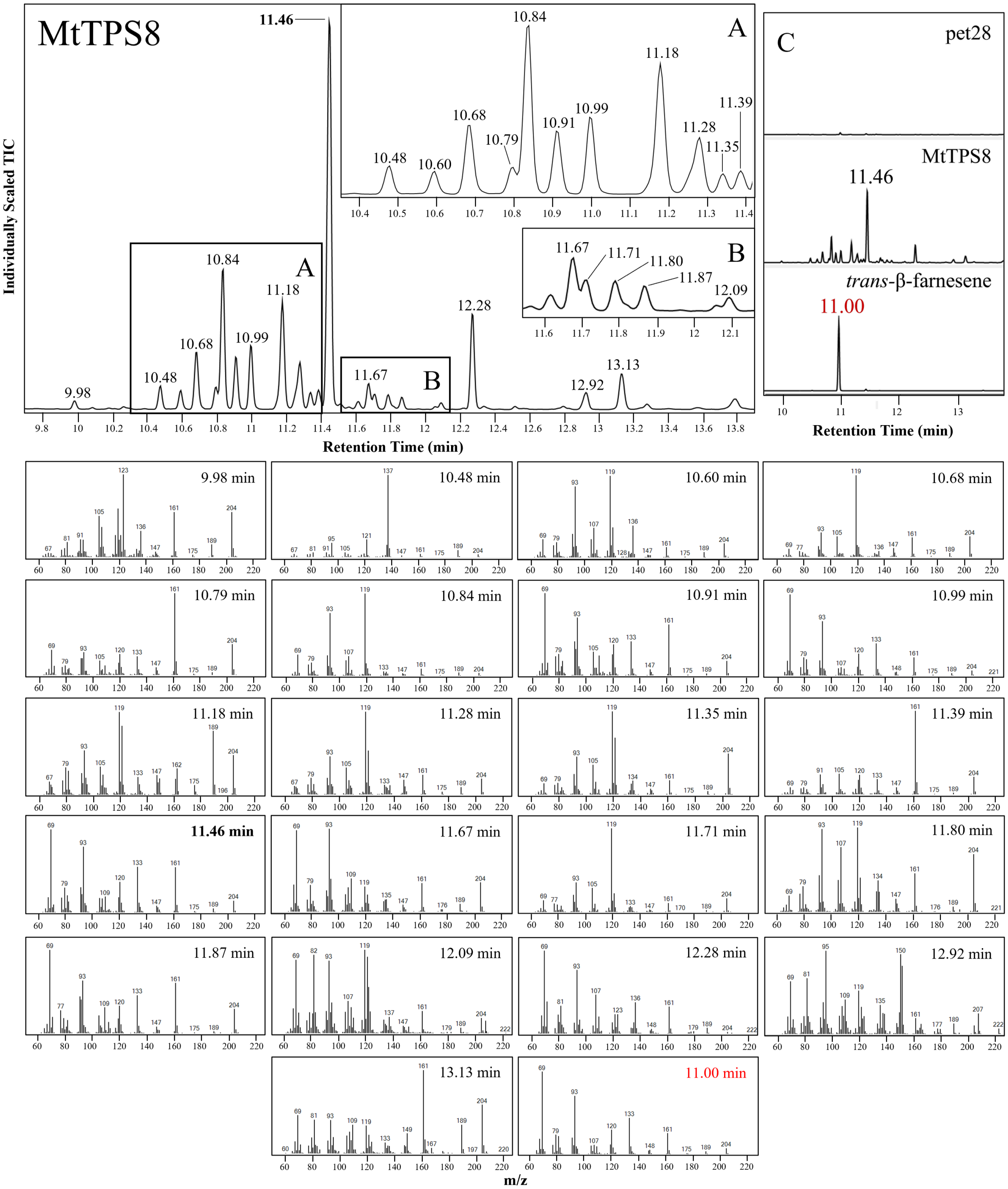

MtTPS8 C15 Peaks					
9.98 min	(+)-cyclosativene	10.99 min	cis-β-farnesene	11.71 min	β-curcumene
10.48 min	petasitene	11.18 min	amorpha-4,11-diene	11.80 min	cis-γ-bisabolene
10.60 min	cedr-8-ene	11.28 min	4-epi-α-acoradiene	11.87 min	β-sesquiphellandrene
10.68 min	cedr-8-ene	11.35 min	γ-curcumene	12.09 min	cis-sesquisabinene hydrate
10.79 min	β-cedrene	11.39 min	β-cubebene	12.28 min	(+)-trans-nerolidol
10.84 min	α-bergamotene	**11.46 min**	**farnesene-like**	12.92 min	cedrol
10.91 min	β-sesquiphellandrene	11.67 min	β-bisabolene	13.13 min	agarospirol

Figure S4: GC-MS of minor compounds produced by MtTPS8.
In addition to its main farnesene-like product, which has an RT of 11.46 min and is highlighted above, *MtTPS8* produced approximately twenty putative sesquiterpene products. Mass spectra correspond to labelled peaks (which are labelled according to their retention times) and best identity matches from the NIST database are listed below. Smaller peaks are shown in greater detail in panels **A** and **B**, which correspond to the similarly labelled area of the GC spectrum. The *cis*-β-farnesene peak was also seen in the control sample at 11.0 minutes and is not an *MtTPS8* product. Panel **C** shows comparison of the *MtTPS8* farnesene-like product to a *trans*-β-farnesene standard which showed a difference in retention times. The MS spectra for *trans*-β-farnesene standard is labeled in red and is a match for the *MtTPS8* product. The *Mt*TPS8 product is a match by mass spectra to *trans*-β-farnesene but as it does not match its retention time it is not a confirmed match and the *MtTPS8* product is considered farnesene-like.

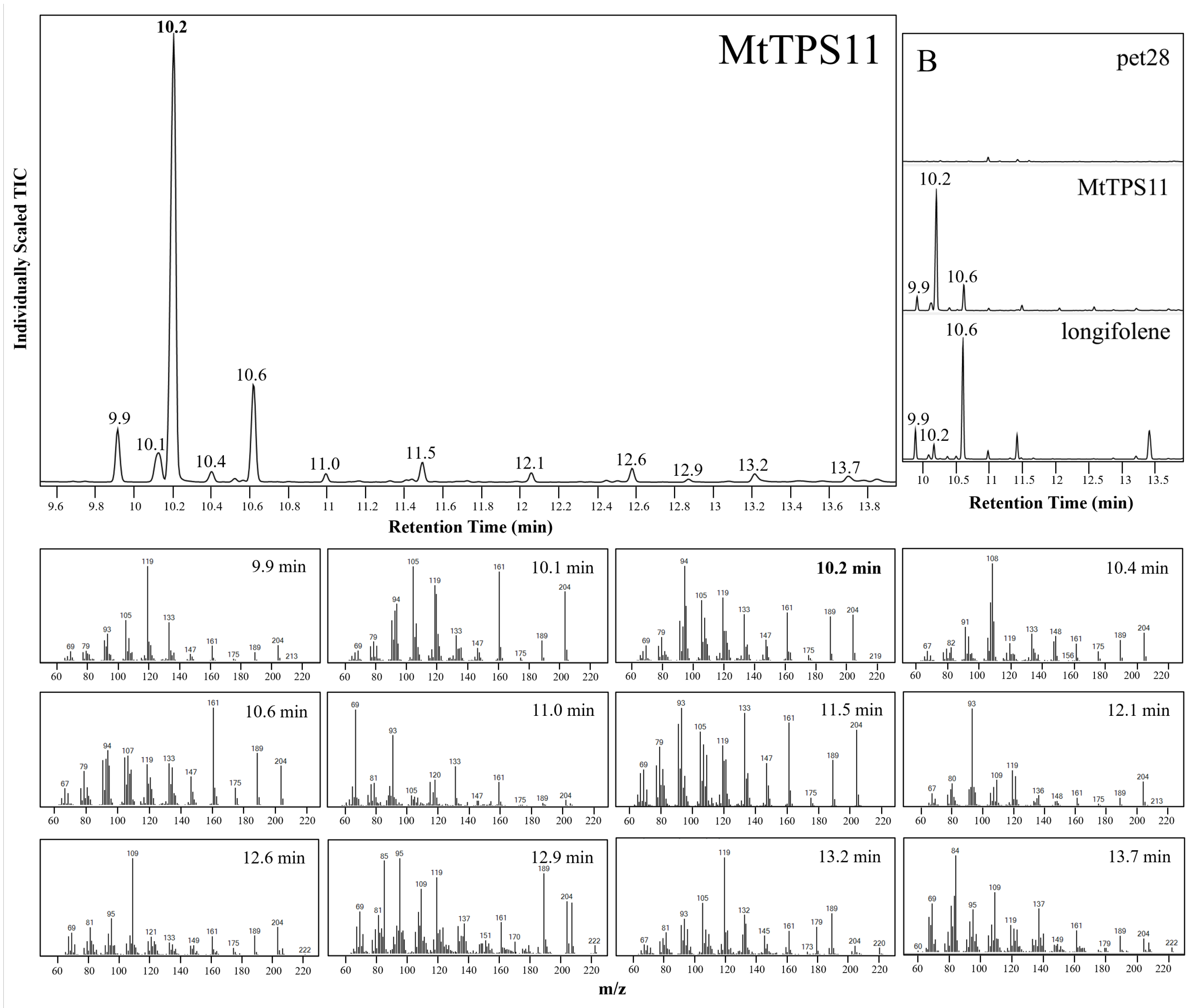

MtTPS11 C15 Peaks					
9.9 min	α-longipinene	10.6 min	(+)-longifolene	12.6 min	humulane-1,6-dien-3-ol
10.1 min	(+)-cyclosativene	11.0 min	cis-β-farnesene	12.9 min	longiborneol
**10.2 min**	**longicyclene**	11.5 min	caryophyllene	13.2 min	cedr-8-en-13-ol
10.4 min	α-bulnesene	12.1 min	trans-α-bisabolene	13.7 min	shyobunol

Figure S5: GC-MS of minor compounds produced by MtTPS7.

*Mt*TPS11 creates several C15 products in addition to its main product longicyclene, which has an RT of 10.2 min and is highlighted above. MS spectra correspond to labelled peaks (which are labelled according to their retention times) and best identity matches from the NIST database are listed below. The *cis*-β-farnesene peak was also seen in the control sample at 11.0 minutes and is not an *MtTPS11* product. Panel **B** shows a comparison of MtTPS11 products to the longifolene standard *PaTPS-lon* which also makes longicyclene and α-longipinene in small amounts.

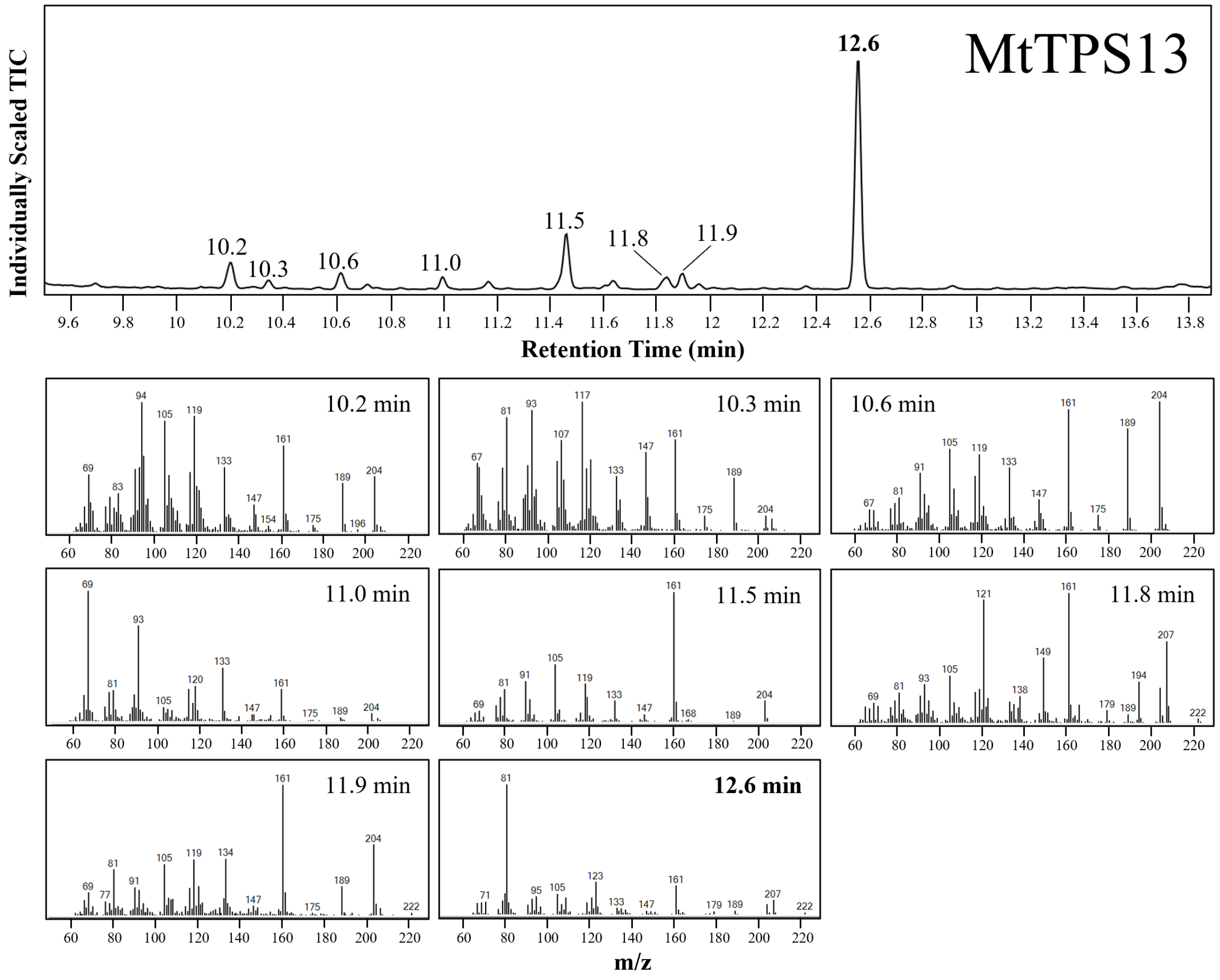

MtTPS13 C15 Peaks			
10.2 min	longicyclene	11.5 min	cis-β-copaene
10.3 min	β-elemene	11.8 min	cubebol
10.6 min	α-gurjunene	11.9 min	β-cadinene
11.0 min	cis-β-farnesene	**12.6 min**	**germacrene D-4-ol**

Figure S6. Additional peak labelling for *MtTPS13* expression. *MtTPS13* creates several C15 products in addition to its main product germacrene D-4-ol, which has an RT of 12.6 min and is highlighted above. MS spectra correspond to labelled peaks (which are labelled according to their retention times) and best identity matches from the NIST database are listed below. The *cis*-β-farnesene peak was also seen in the control sample at 11.0 minutes and is not an *MtTPS13* product.

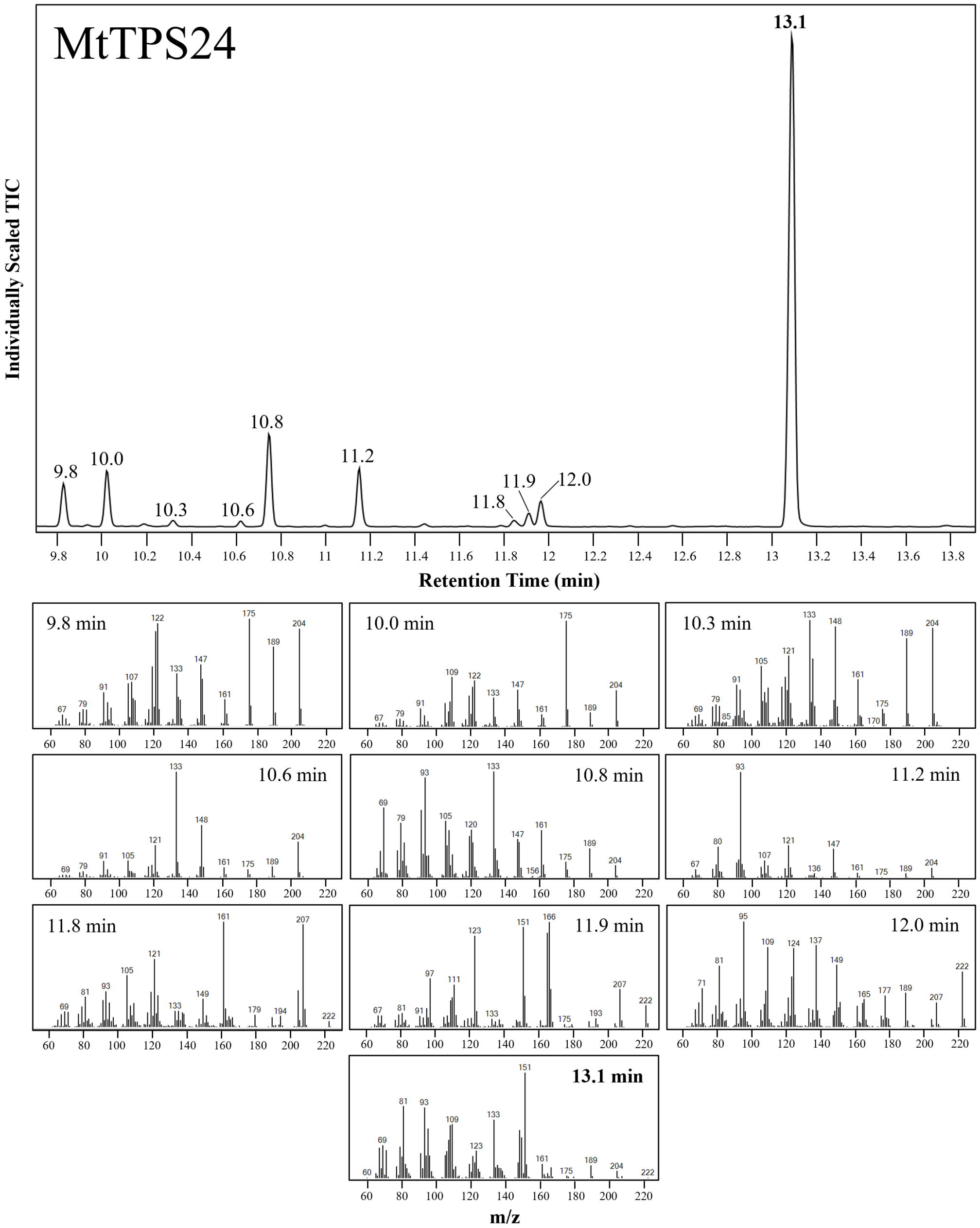

MtTPS24 C15 Peaks			
9.8 min	7-epi-silphperfol-5-ene	11.2 min	humulene
10.0 min	7-epi-silphperfol-5-ene	11.8 min	cubebol
10.3 min	unknown C15 product	11.9 min	unknown C15 product
10.6 min	unknown C15 product	12.0 min	unknown C15 product
10.8 min	caryophyllene	**13.1 min**	**unknown C15 product**

Figure S7: GC-MS of minor compounds produced by MtTPS24.
*Mt*TPS24 forms C15 products in addition to its main unknown product, which has an RT of 13.1 min and is highlighted above. MS spectra correspond to labelled peaks (which are labelled according to their retention times), and best identity matches from the NIST database are listed below.

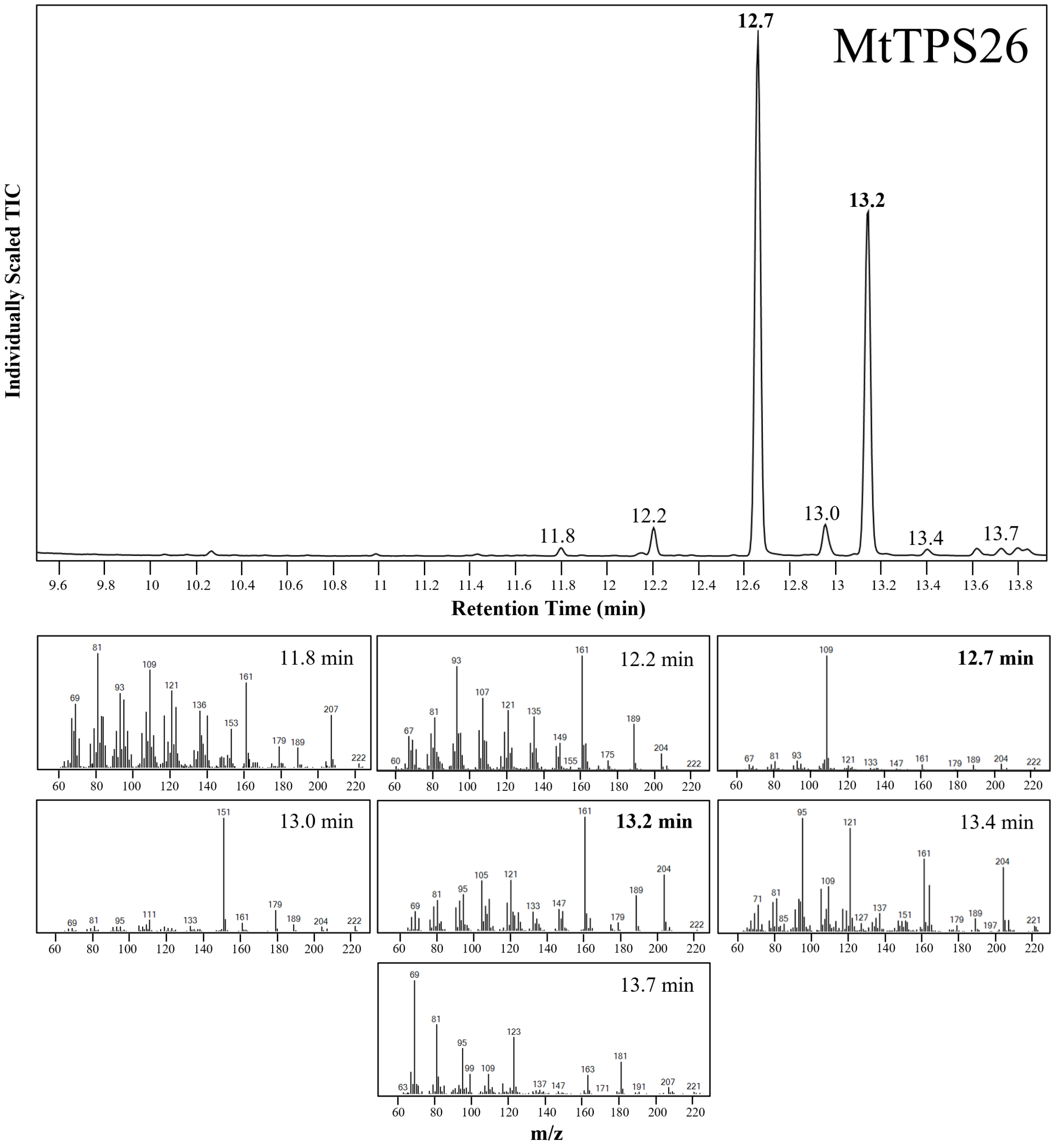

MtTPS26 C15 Peaks			
11.8 min	shyobunol	**13.2 min**	**guai-6-en-10β-ol**
12.2 min	cadinol-like	13.4 min	α-cadinol
**12.7 min**	**eudesm-4(14)-en-6β-ol**	13.7 min	globulol
13.0 min	widdrol		

Figure S8: GC-MS of minor compounds produced by MtTPS26.
*Mt*TPS26 forms several C15 products in addition to its main products, which have RTs of 12.7 and 13.2 min, respectively, and are highlighted above. MS spectra correspond to labelled peaks (which are labelled according to their retention times) and best identity matches from the NIST database are listed below.

Table S4: Summary of metabolites produced by TPS-a family members in Arabidopsis, *S. lycopersicum* and *M. truncatula*

NOTE: The table is an adaptation from Zhou and Pichersky (2020) New Phytologist. The complete functional characterization of terpene synthase family in tomato.
The M. truncatula metabolites are a summary compiled from Figures 1 and 4 in the main text (Ref #'s 6, 7, 9 &32).

Synthase	Phytozome ID	Substrate	Characterized Product(s)
Arabidopsis thaliana			
AtTPS01	*AT4G15870*		Unknown
AtTPS05	*AT2G23230*		Unknown
AtTPS06	*AT1G70080*	(*E*, *E*, *E*)-GGPP	Dolabelladienol
AtTPS07	*AT4G20200*		Unknown
AtTPS08	*AT4G20210*	(*E*, *E*, *E*)-GGPP	Rhizathalene A
AtTPS09	*AT4G20230*	(*E*, *E*, *E*)-GGPP	Unidentified
AtTPS11	*AT5G44630*	(*E*, *E*)-FPP	Multiproduct
AtTPS12	*AT4G13280*	(*E*, *E*)-FPP	γ-Bisabolene
AtTPS13	*AT4G13300*	(*E*, *E*)-FPP	γ-Bisabolene
AtTPS15	*AT3G29190*		Unknown
AtTPS16	*AT3G29110*		Unknown
AtTPS17	*AT3G14490*	GFPP	Multiproduct
AtTPS18	*AT3G14520*	GFPP	(+)-Thalianatriene
AtTPS19	*AT3G14540*	GFPP	(-)-Retigeranin B
AtTPS20	*AT5G48110*	(*E*, *E*, *E*)-GGPP	Dolathaliatriene Dolabelladienol
AtTPS21	*AT5G44630*	(*E*, *E*)-FPP	β-Caryophyllene<br>α-Humulene
AtTPS22	*AT1G33750*	(*E*, *E*)-FPP	β-Farnesene<br>α-Farnesene
AtTPS25	*AT3G29410*	GFPP	(-)-*ent*-Quiannulatene, (-)-Variculatriene A
AtTPS26	*AT1G66020*	(*E*, *E*, *E*) - GGPP	Unidentified
AtTPS28	*AT1G48800*		Unknown
AtTPS29	*AT1G31950*		Unknown
AtTPS30	*AT3G32030*	GFPP	(+)-Astallatene
Solanum lycopersicum			
SlTPS9	*Solyc06g059885*	(*E*, *E*)-FPP<br>(*Z*, *Z*)-FPP	Germacrene C
SlTPS10	*Solyc06g059910*<br>*Solyc06g059920*	(*Z*, *Z*)-FPP	α-bisabolol
SlTPS12	*Solyc06g059930*	(*E*, *E*)-FPP<br>(*Z*, *Z*)-FPP	β-caryophyllene<br>α-Humulene/Curcumene
SlTPS14	*Solyc09g092470*	(*E*, *E*)-FPP<br>(*Z*, *Z*)-FPP	β-bisabolene<br>α-bisabolene
SlTPS16	*Solyc07g008680*<br>*Solyc07g008690*	(*E*, *E*)-FPP	δ-cadinene
SlTPS17	*Solyc12g006570*	(*E*, *E*)-FPP<br>(*Z*, *Z*)-FPP	Valencene<br>γ-Bisabolene
SlTPS28	*Solyc04g054380*	(*E*, *E*)-FPP<br>(*Z*, *Z*)-FPP	Hedycaryol<br>unidentified
SlTPS31	*Solyc01g101170*	(*E*, *E*)-FPP	Viridiflorene
SlTPS32	*Solyc01g101180*	(*E*, *E*)-FPP	Viridiflorene

		(*Z, Z*)-FPP	Unidentified
SlTPS33	*Solyc01g101190*	(*E, E*)-FPP<br>(*Z, Z*)-FPP	Guaia-1(10),11-diene<br>β-Acoradiene
SlTPS35	*Solyc01g101210*	(*E, E*)-FPP<br>(*Z, Z*)-FPP	Guaia-1(10)-,11-diene<br>(*Z, Z*)-farnesol
SlTPS36	*Solyc06g060180*	(Z, *Z*)-FPP	*Cis*-Muurola-3,5-diene
SlTPS48	*Solyc04g051620*	(*E, E*)-FPP<br>(*Z, Z*)-FPP	Hedycaryol<br>unidentified
SlTPS51	*Solyc7g052120*<br>*Solyc7g052130*	(*E, E*)-FPP<br>(*Z, Z*)-FPP	*E*-Nerolidol<br>α-Bisabolol
SlTPS52	*Solyc07g052140*<br>*Solyc07g052150*	(*E, E*)-FPP<br>(*Z, Z*)-FPP	*E*-Nerolidol<br>α-Bisabolol
Medicago truncatula			
**Synthase**	**Phytozome ID**	**Substrate**	**Characterized Product(s)**
MtTPS1	*Medtr4g081460*	(*E, E*)-FPP<br>GPP	β - Caryophyllene<br>Myrcene, Limonene, Terpinolene, uniidentified
MtTPS2	*Medtr6g039440*	(*E, E*)-FPP	No products detected
MtTPS5	*Medtr5g062230*	(*E, E*)-FPP	Cubebol (multiproduct synthase)
		(*Z, E*)-FPP	δ-Amorphene, humula-4,9-dien-8-ol<br>(multiproduct synthase)
		GPP	Terpinolene (multiproduct synthase)
MtTPS7	*Medtr2g081980*	(*E, E*)-FPP	Cylclosativene; α-copaene
MtTPS8	*Medtr2g082010*	(*E, E*)-FPP	Farnesene (multiproduct synthase)
MtTPS9	*Medtr2g082060*	(*E, E*)-FPP	No products detected
MtTPS10	*Medtr5g073200*	(*E, E*)-FPP	Himachalol
MtTPS11	*Medtr5g073260*	(*E, E*)-FPP	Longicyclene
MtTPS12	*Medtr5g094620*	(*E, E*)-FPP	No products detected
MtTPS13	*Medtr6g008560*	(*E, E*)-FPP	Germacrene-D-4-ol
MtTPS24	*Medtr3g464190*	(*E, E*)-FPP	Unidentified
MtTPS25	*Medtr4g048460*	(*E, E*)-FPP	No products detected
MtTPS26	*Medtr8g007515*	(*E, E*)-FPP	Eudesm4(14)-en-6β-ol &<br>Guai-6-en-10β-ol
MtTPS27	*Medtr4g019550*	(*E, E*)-FPP	No products detected

Table S5: ^{13}C and ^{1}H-NMR spectroscopic data of compound 8

Position	^{1}H (multiplicity, *J* in Hz)	^{13}C	Main HMBC correlations
**1**	1,77 (1H, td, 13.4, 4.5)<br>1.01 (1H, dtt, 13.3, 3.8, 1.5 Hz,)	31.1 (t)	C-5, C-10, C-3, C-2
**2**	1.65 (2H, ddt, 13.2, 9.1, 4.7)	23.0 (t)	C-10, C-3
**3**	2,17 (2H, m)	30.4 (t)	C-2, C-1, C-4, C-5, C-15
**4**	-	147.6 (s)	
**5**	1.70 (1H, d, 9.8)	60.7 (d)	C-4, C-15, C-6, C-10, C-3, C-7
**6**	3,54 (t, J = 9.9 Hz, 1H)	67.9 (d)	C-11, C-4, C-5, C-8, C-7
**7**	1,25 (1H, m)	49.3 (d)	C-6, C-7, C-12, C-10, C-11, C-8
**8**	1,29 (1H, m)<br>1.46 – 1.39 (1H, m)	18.7 (t)	C-6, C-7, C-9, C-10, C-1, C-14, C-8
**9**	1,29 (1H, m)<br>1.51 – 1.47 (1H, m)	40.2 (t)	C-6, C-1, C-7, C-8, C-10
**10**	-	35.8 (s)	
**11**	2.23 (1H, pt, 8.8, 4.3)	26.6 (d)	C-6, C-7, C-12, C-13, C-8
**12**	0.96 (3H, d, 7.0)	21.1 (q)	C-7, C-13, C-11
**13**	0.87 (3H, d, 6.9)	16.4 (q)	C-7, C-12, C-11
**14**	0.92 (3H, s)	28.5 (q)	C-5, C-1, C-9, C-10
**15**	4.81 (1H, t, 2.1)<br>4.96 (1H, t, 2.3)	113.1 (t)	C-3, C-4, C-5

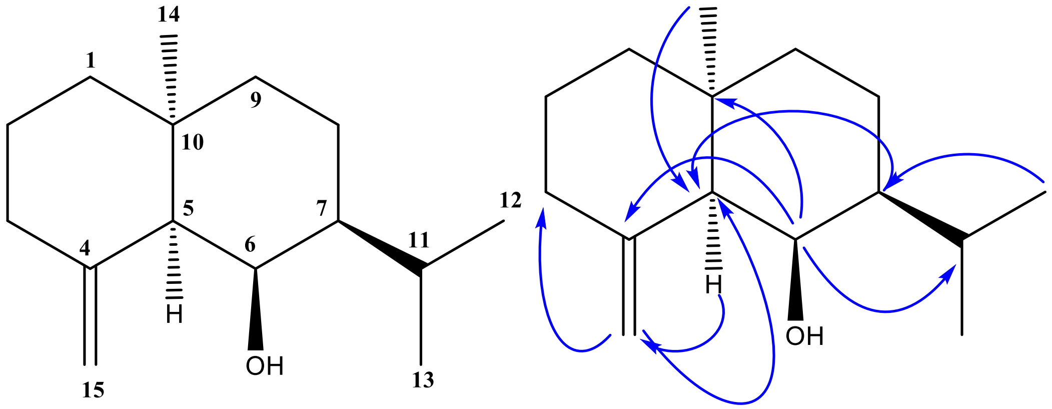

Figure S9: Numbering and main HMBC correlations of compound **8**

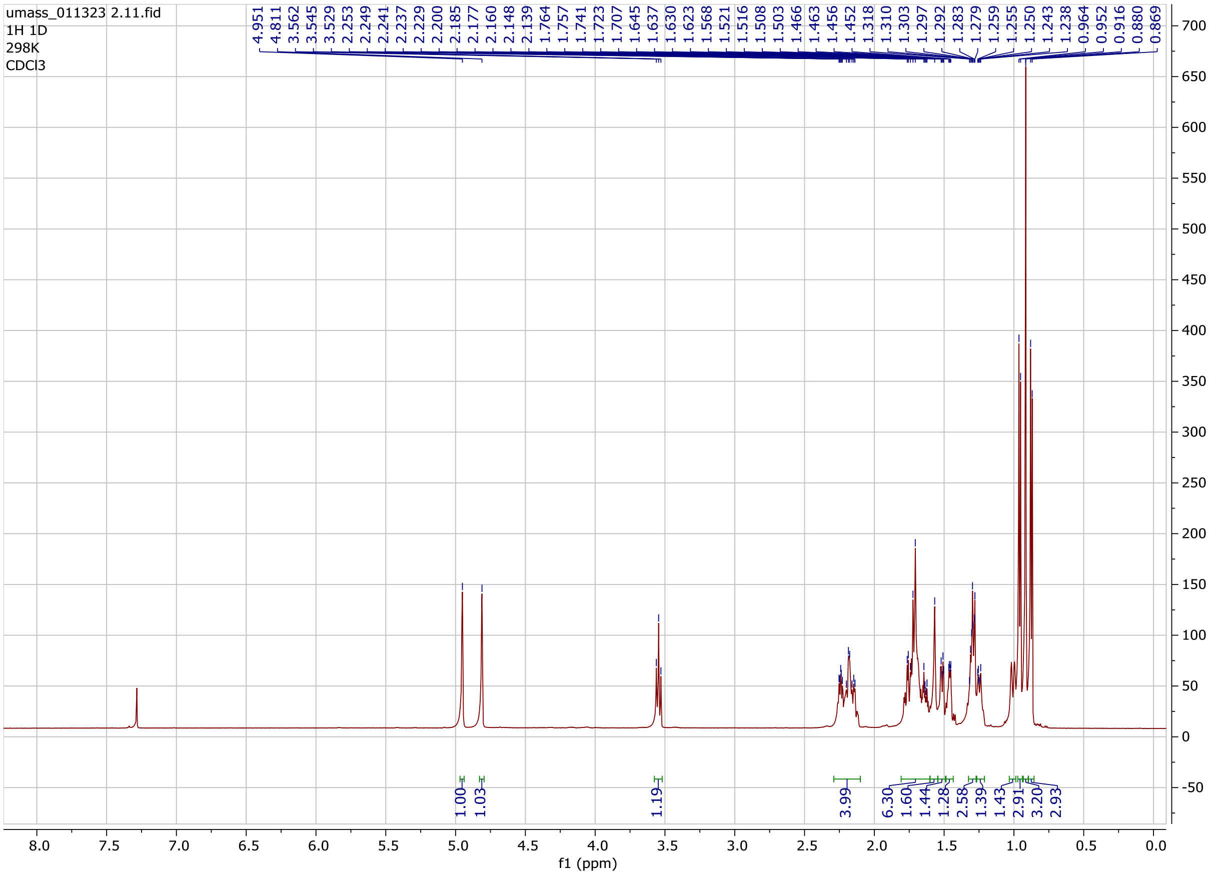

Figure S10a: ^{1}H-NMR spectrum ($CDCl_3$, 500 MHz) of compound **8** (full spectrum)

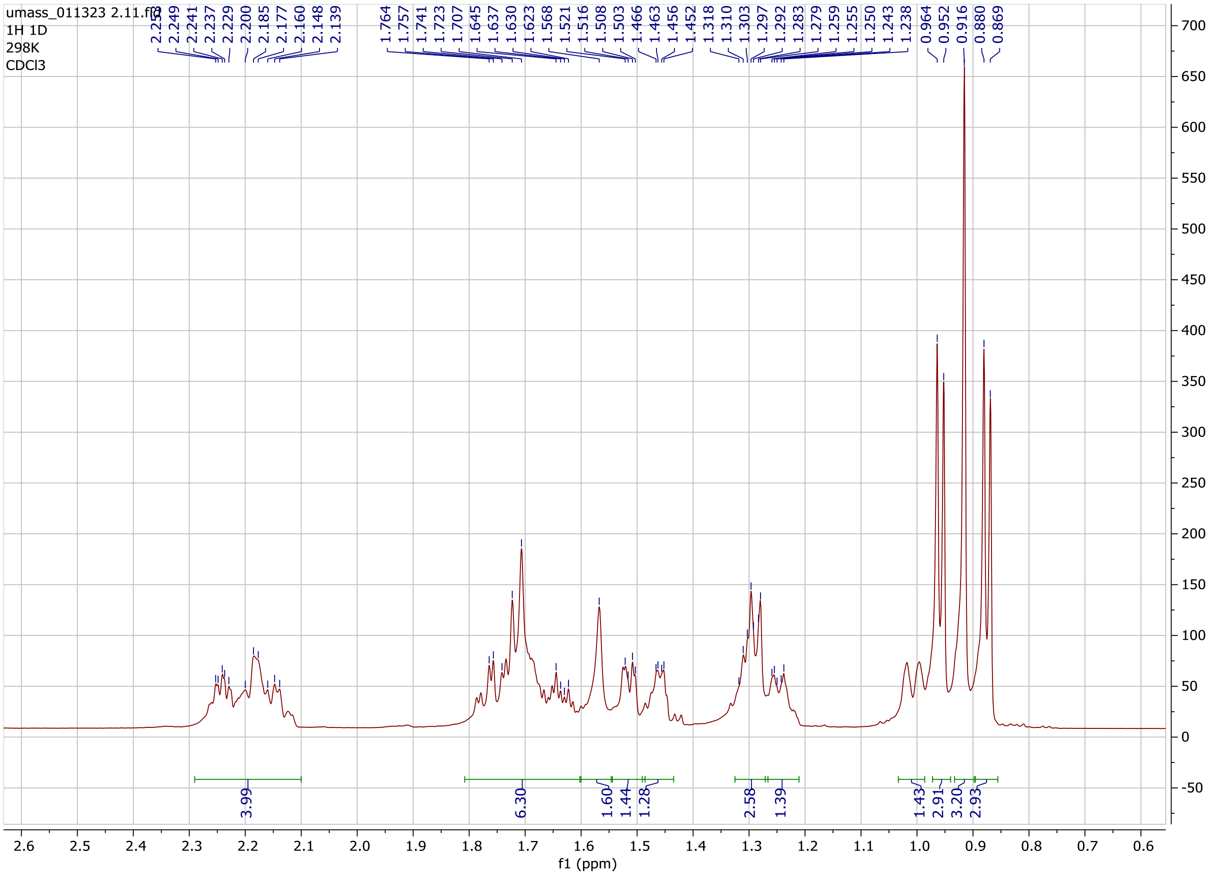

Figure S10b: ^{1}H-NMR spectrum ($CDCl_3$, 500 MHz) of compound **8** (upfield)

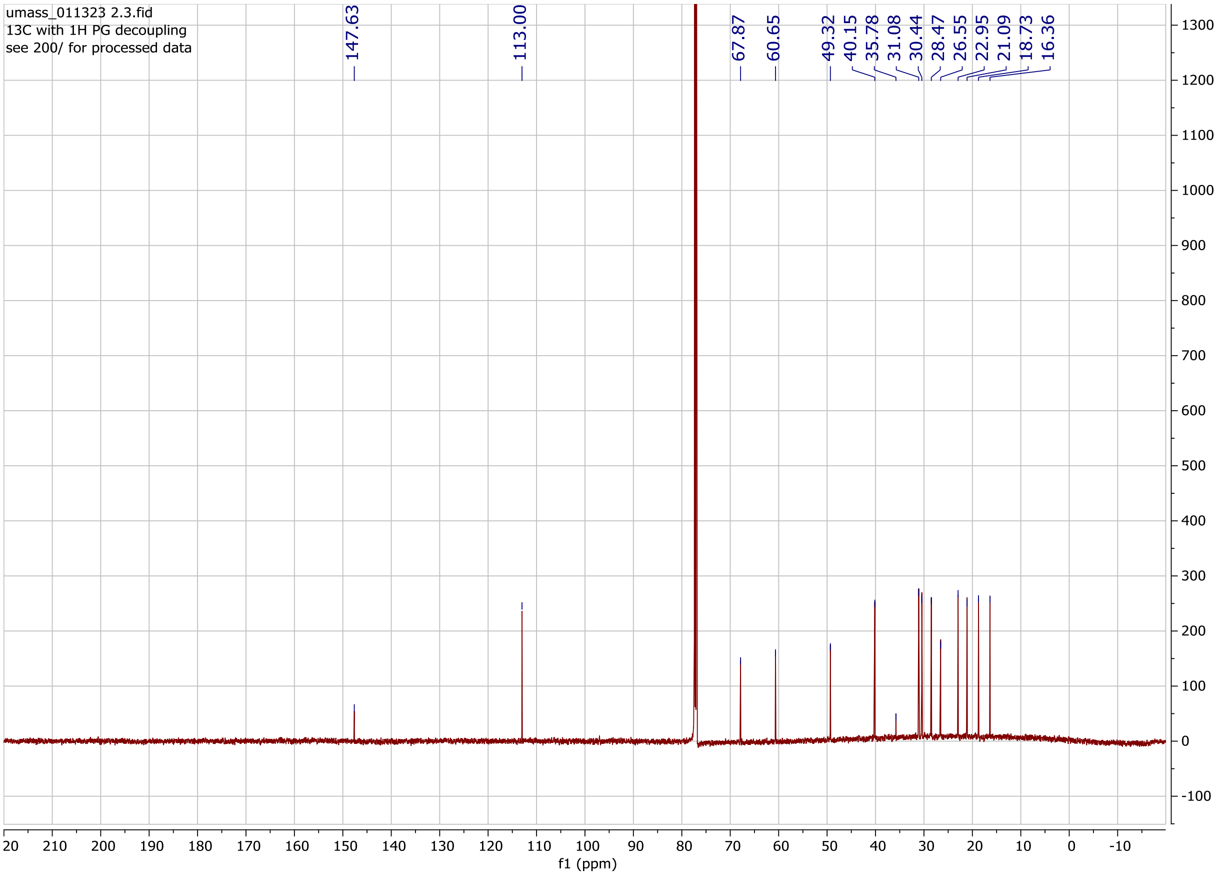

Figure S11: ^{13}C-NMR spectrum ($CDCl_3$, 125 MHz) of compound **8** (full spectrum)

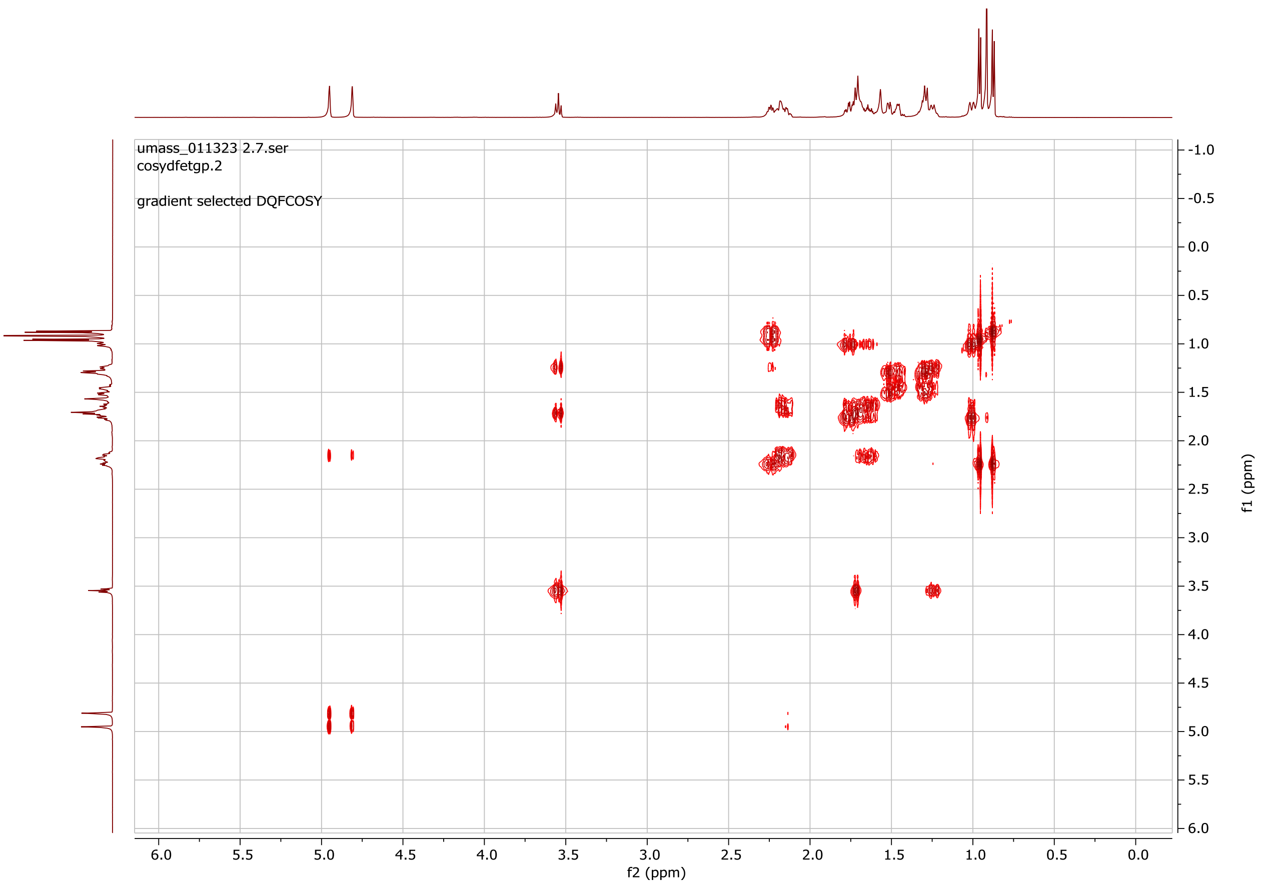

Figure S12: ^{1}H-^{1}H COSY spectrum of compound **8** (full spectrum)

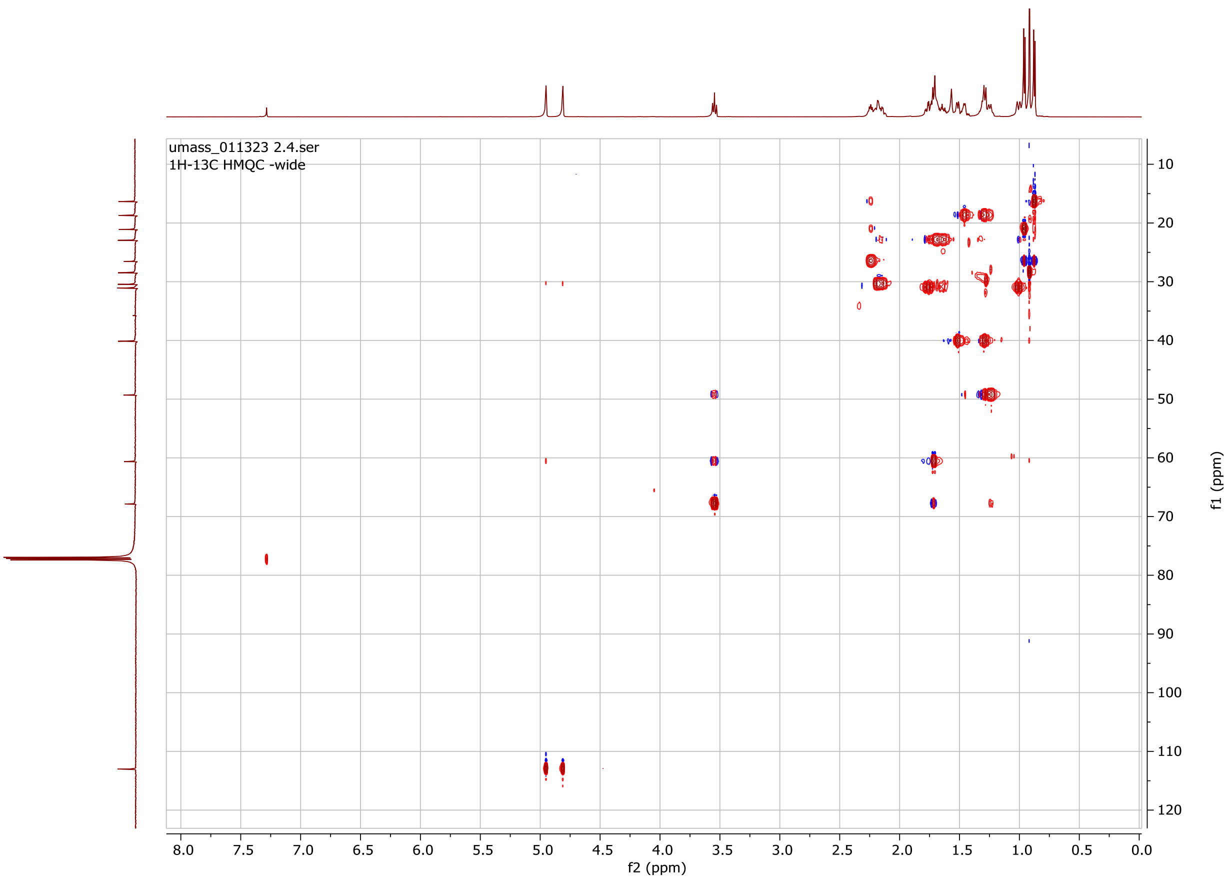

Figure S13: HMQC spectrum of compound **8** (full spectrum)

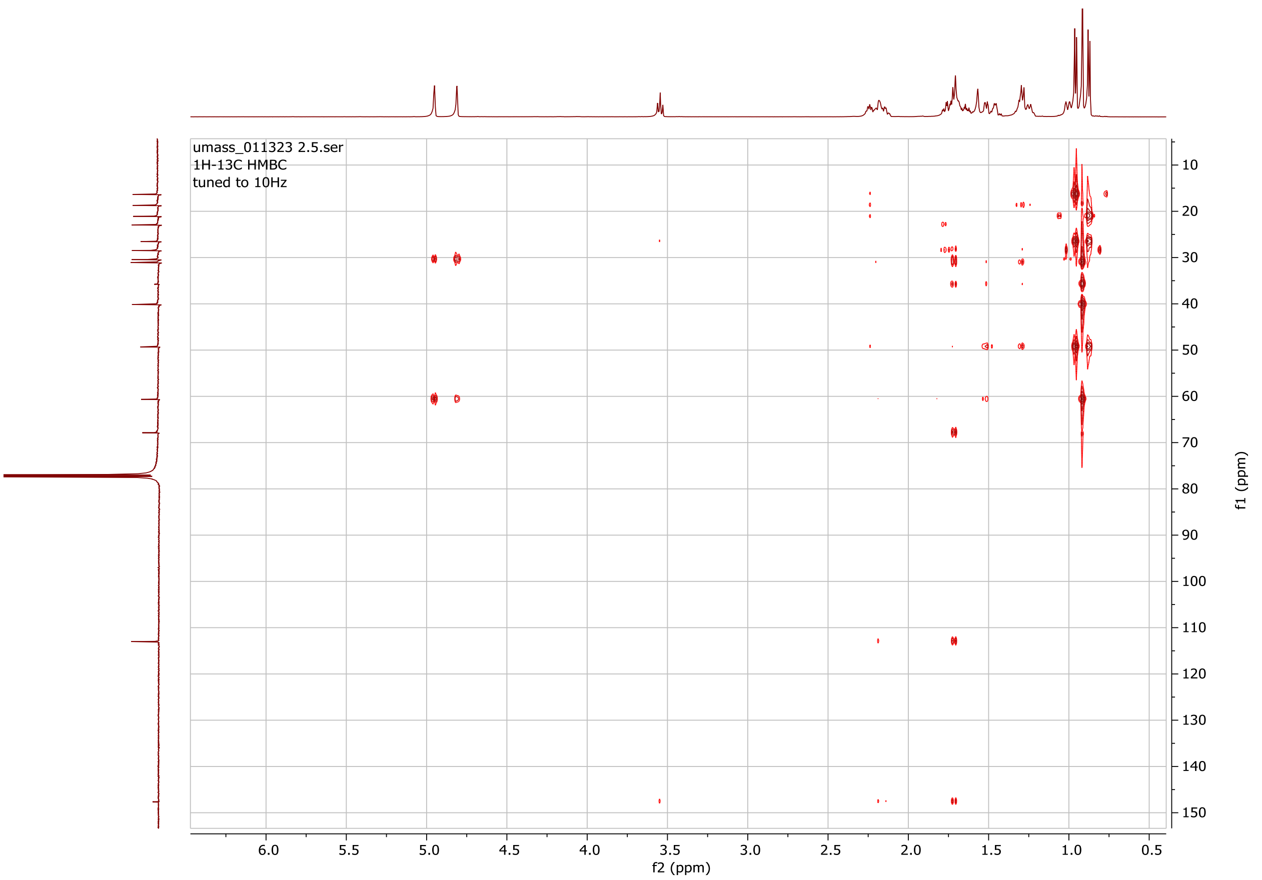

Figure S14a: HMBC spectrum of compound **8** (full spectrum)

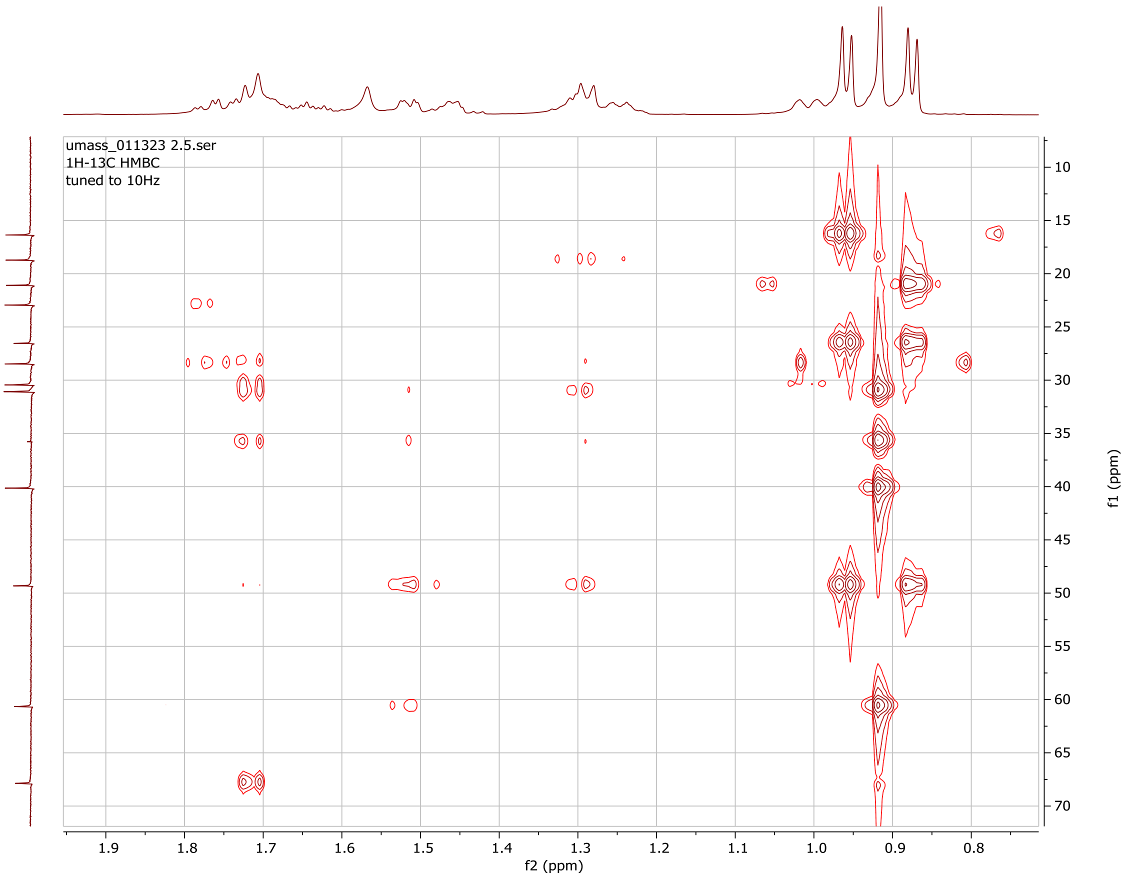

Figure S14b: HMBC spectrum of compound **8** (upfield)

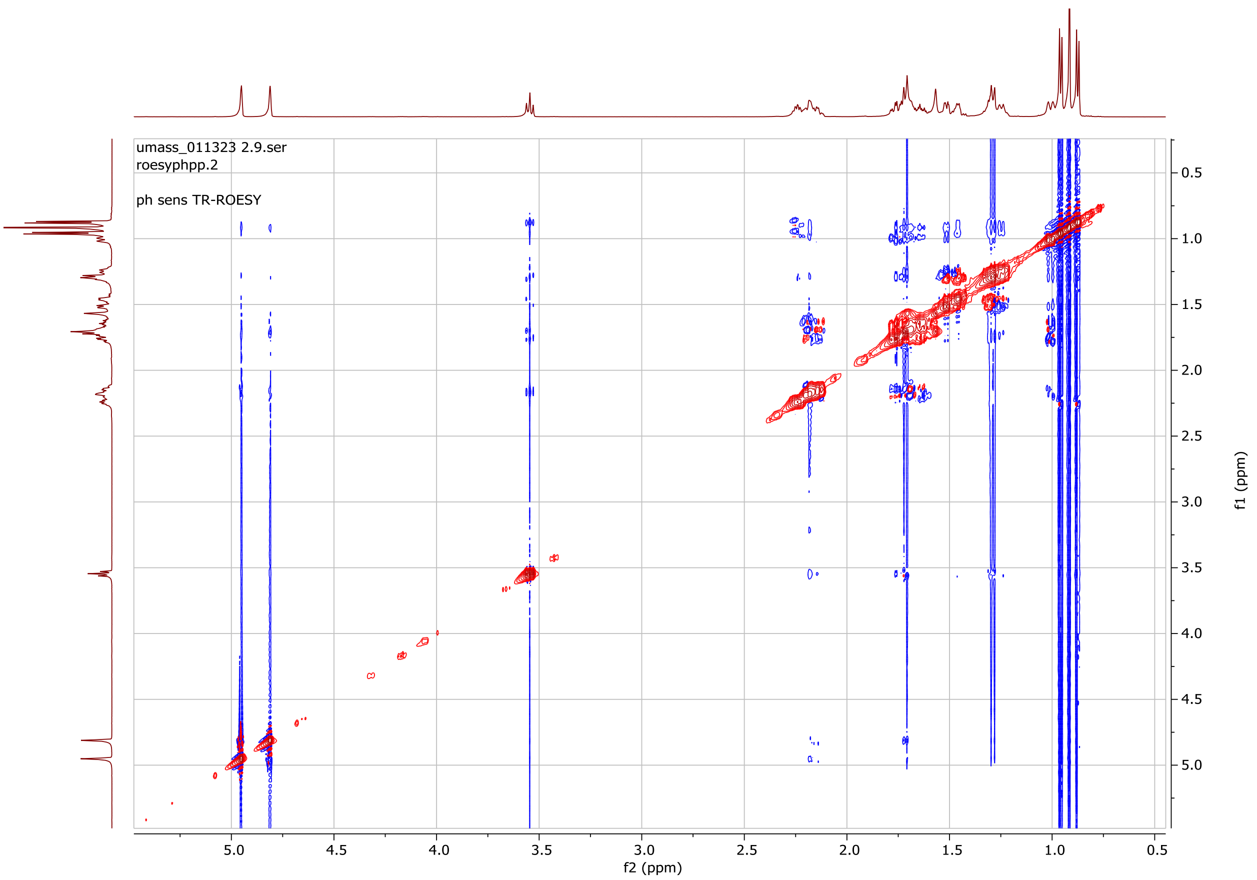

Figure 15: ^{1}H-^{1}H ROESY spectrum of compound **8** (full spectrum)

Table S6: ^{13}C and ^{1}H-NMR spectroscopic data of compound 9

Position	^{1}H (multiplicity, *J* in Hz)	^{13}C	Main HMBC correlations
**1**	1.96 (1H, m)	51.5 (d)	C-5, C-10
**2**	1.83 (1H, m)<br>1.69 (1H, m)	24.1 (t)	
**3**	1.64 (1H, m)<br>1.41 (1H, ddd; 12.6, 8.6, 2.4)	33.3 (t)	C-1, C-4, C-5, C-15
**4**	2.24 (1H, m)	37.8 (d)	
**5**	2.25 (1H, m)	44.0 (d)	
**6**	5.52 (1H, dd; 3.5, 1.2)	124.2 (d)	C-1, C-4, C-5, C-8, C-11
**7**	-	148.4 (s)	
**8**	2.18 (1H, m)<br>1.97 (1H, m)	25.3 (t)	C-6, C-7, C-9, C-10, C-11,
**9**	1.82 (1H, m)<br>1.47 (1H, ddd; 13.0, 10.7, 1.5)	42.8 (t)	C-1, C-7, C-8, C-10
**10**	-	75.7 (s)	
**11**	2.20 (m)	37.3 (d)	
**12**	0.99* (3H, d; 4.5)	21.5 (q)	C-7
**13**	1.00* (3H, d; 4.6)	21.7 (q)	C-7
**14**	1.24 (3H, s)	21.4 (q)	C-1, C-9, C-10
**15**	0.90 (3H, d; 6.9)	15.4 (q)	C-3, C-4, C-5

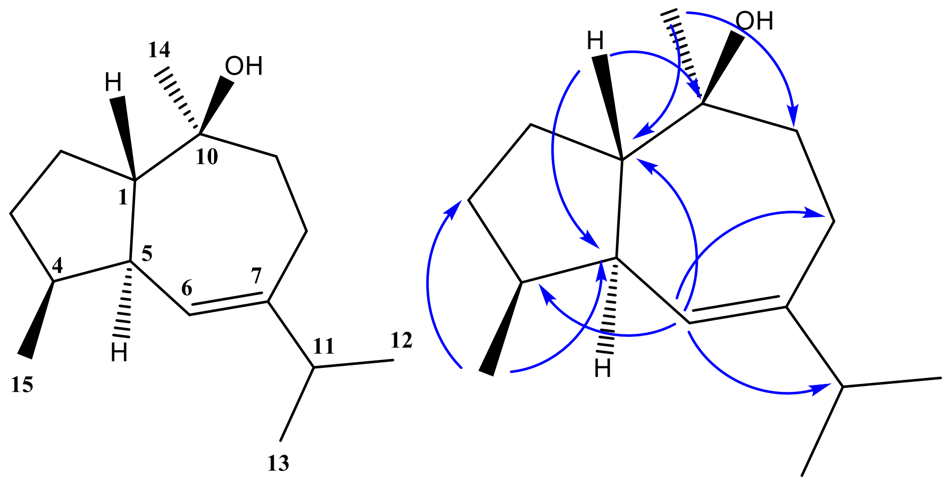

Figure S16: Numbering and main HMBC correlations of compound **9**

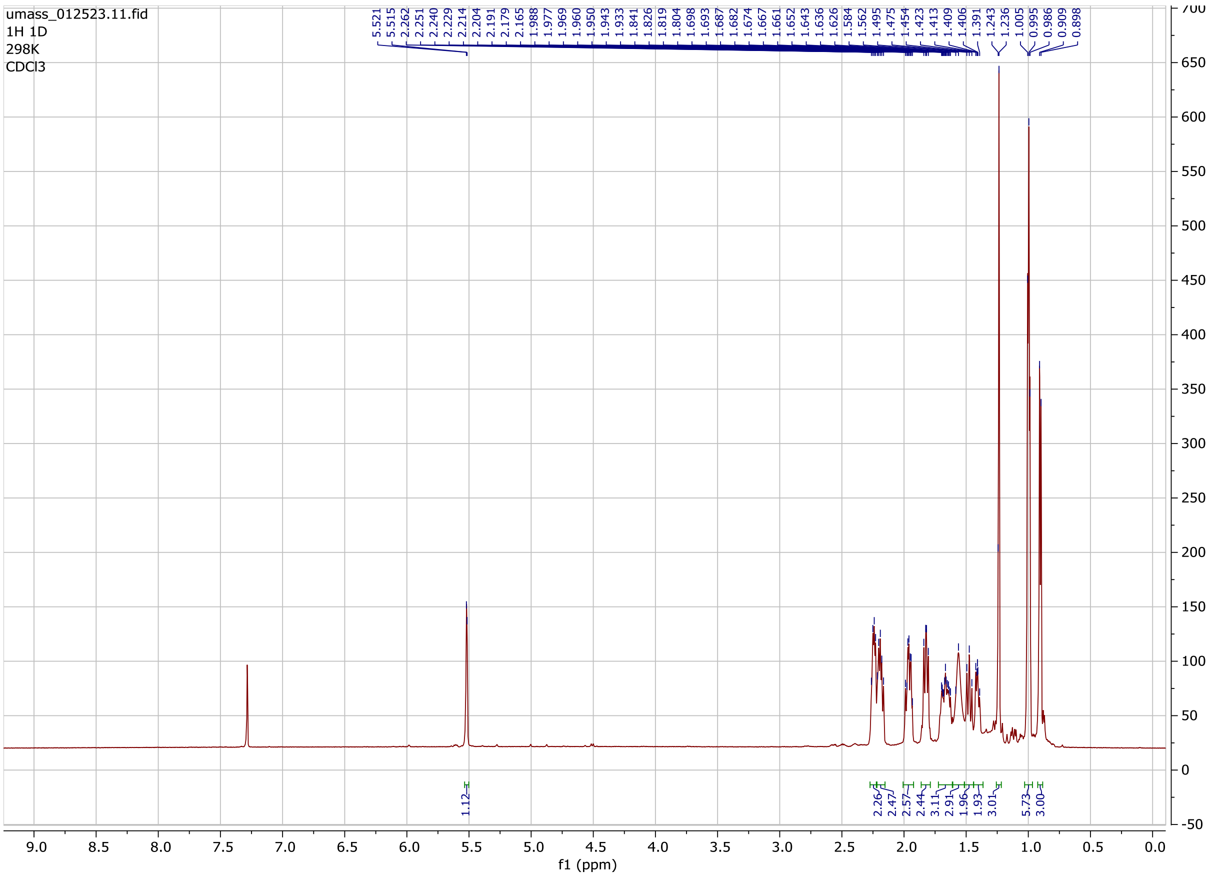

Figure S17a: ^{1}H-NMR spectrum ($CDCl_3$, 500 MHz) of compound **9** (full spectrum)

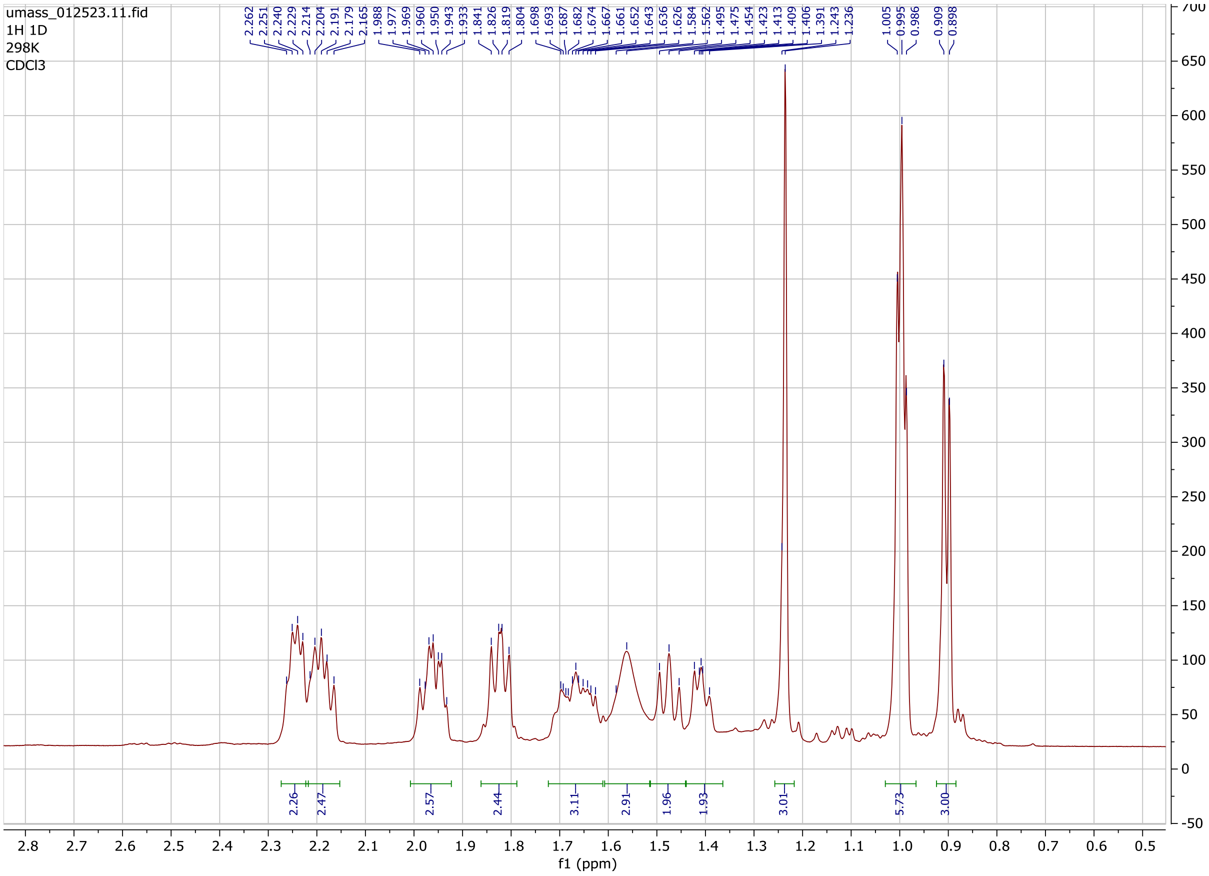

Figure S17b: ^{1}H-NMR spectrum ($CDCl_3$, 500 MHz) of compound **9** (upfield)

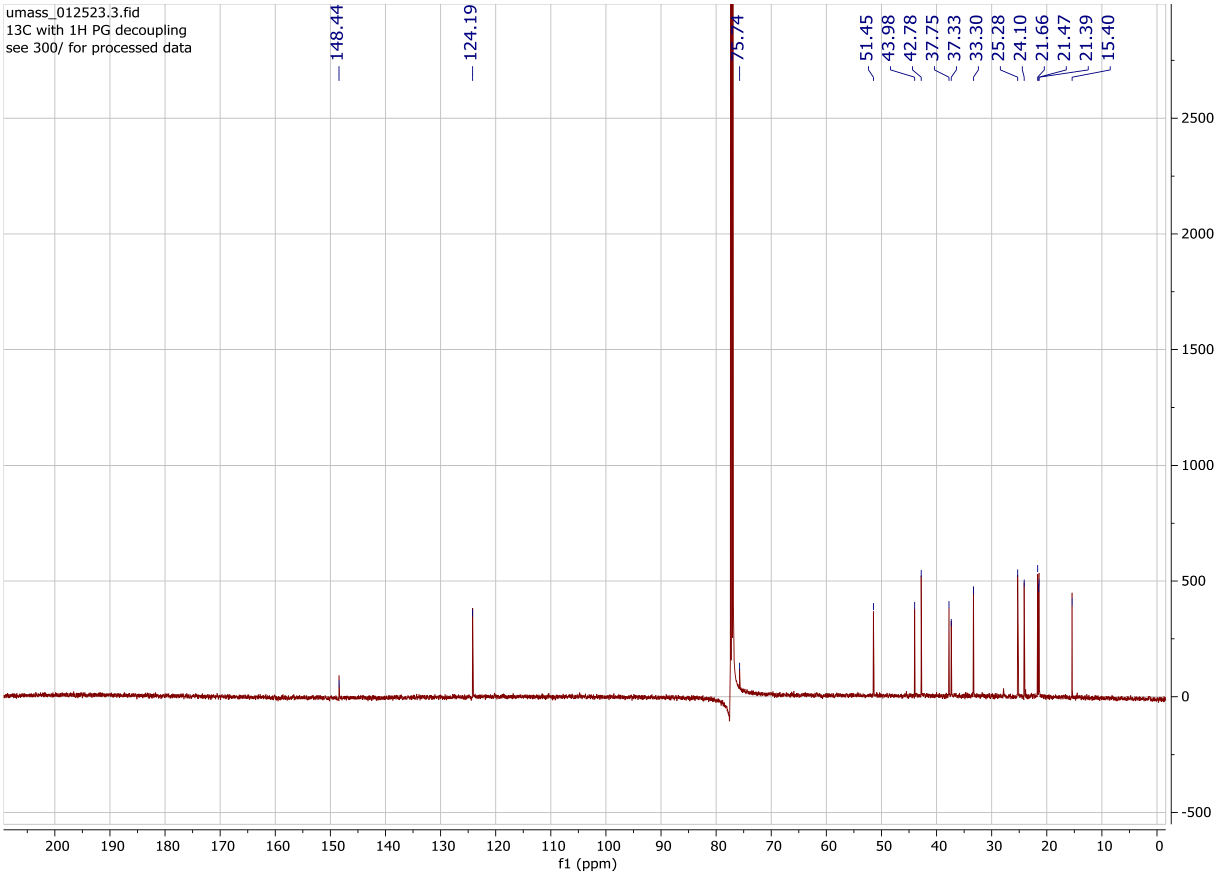

Figure S18: ^{13}C-NMR spectrum ($CDCl_3$, 125 MHz) of compound **9** (full spectrum)

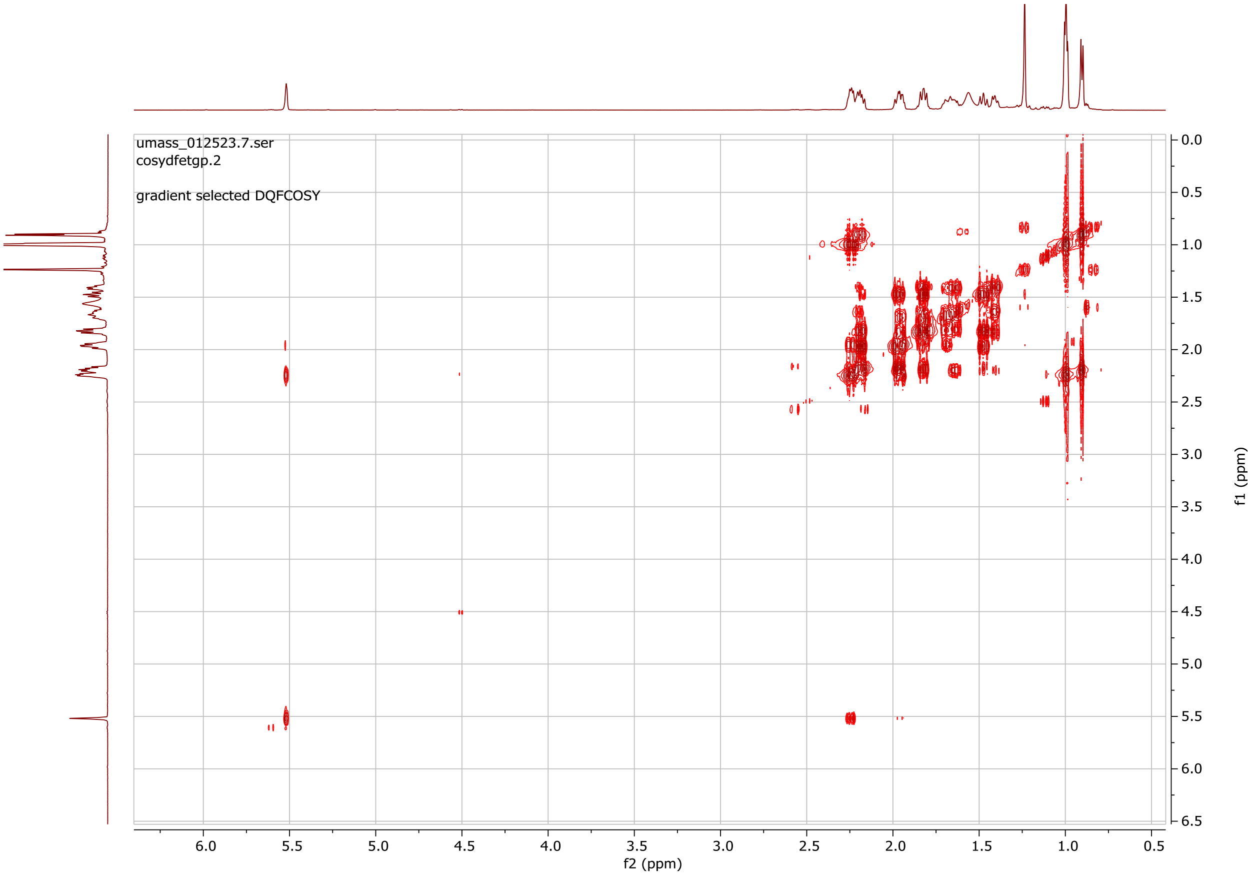

Figure S19a: ^{1}H-^{1}H COSY spectrum of compound **9** (full spectrum)

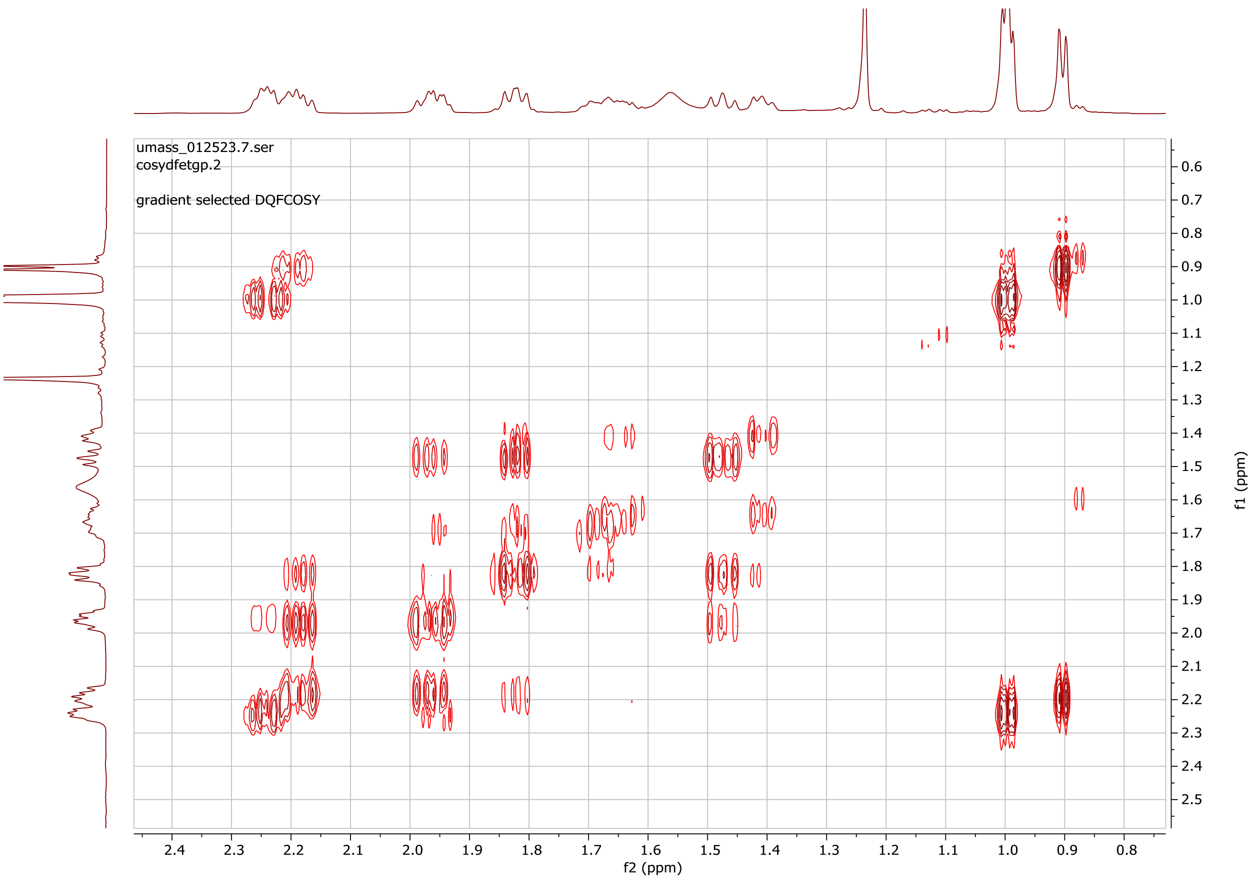

Figure S19b: ^{1}H-^{1}H COSY spectrum of compound **9** (upfield)

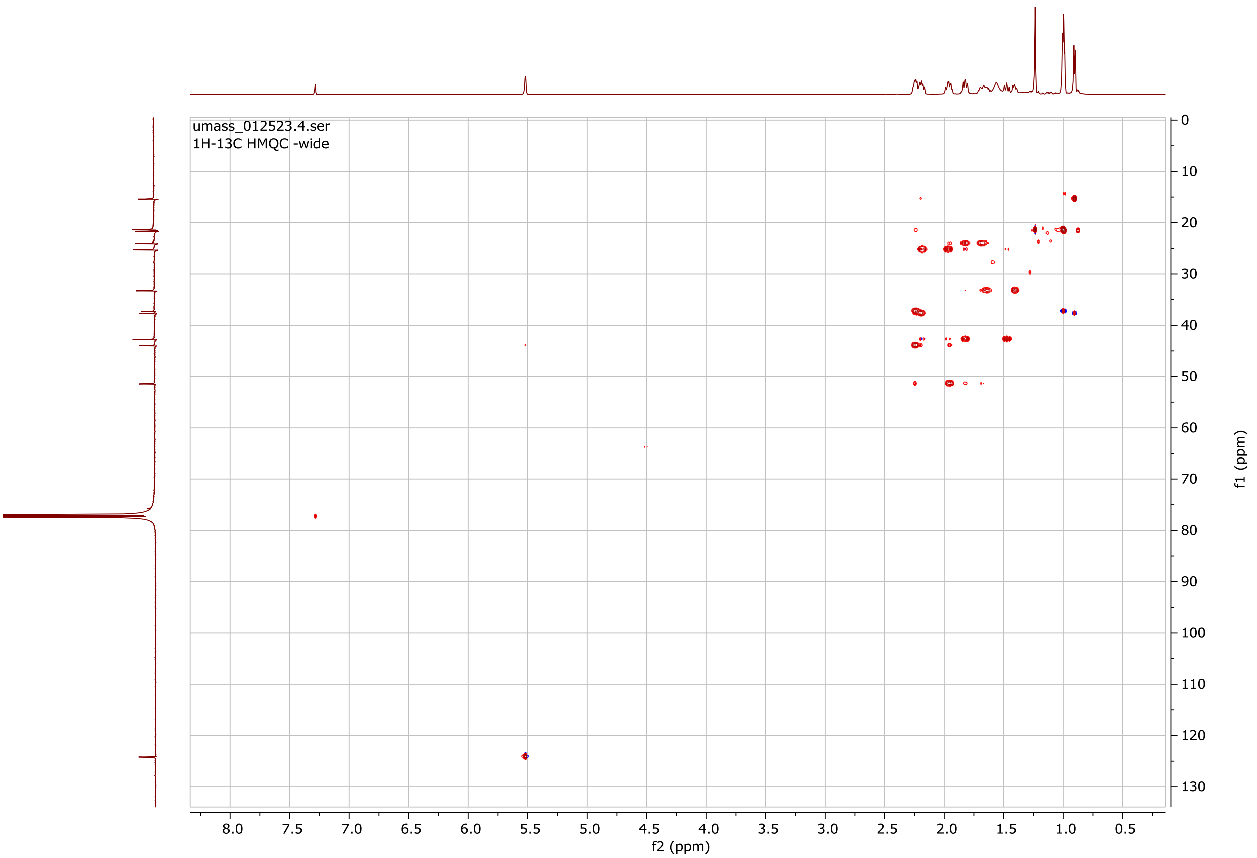

Figure S20a: HMQC spectrum of compound **9** (full spectrum)

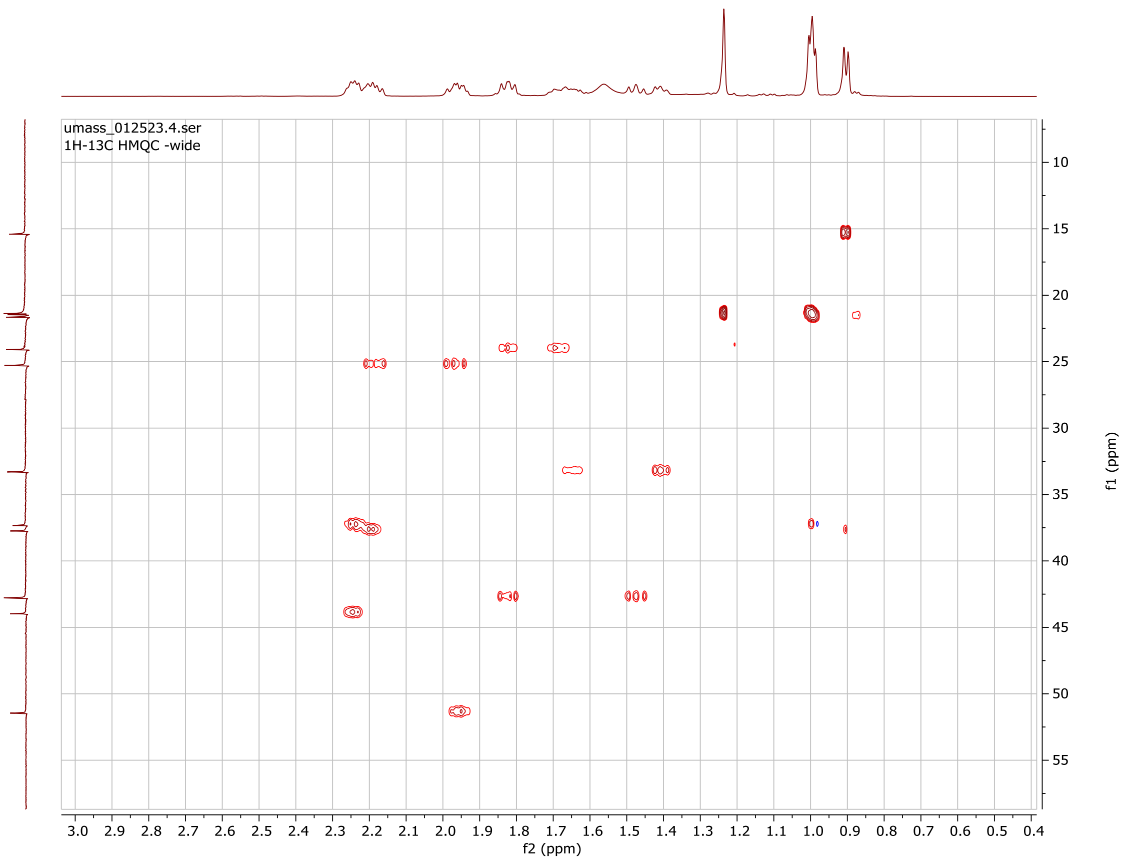

Figure S20b: HMQC spectrum of compound **9** (upfield)

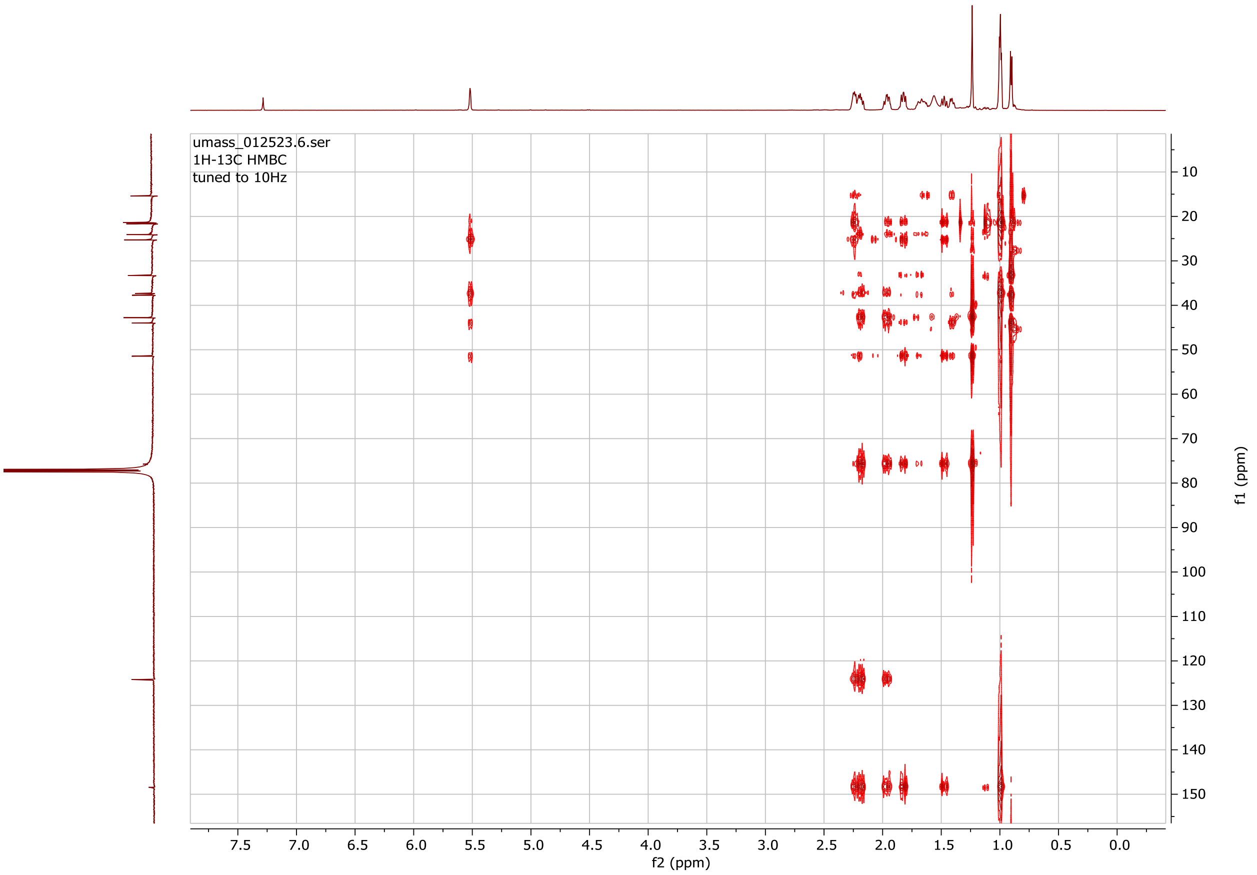

Figure S21a: HMBC spectrum of compound **9** (full spectrum)

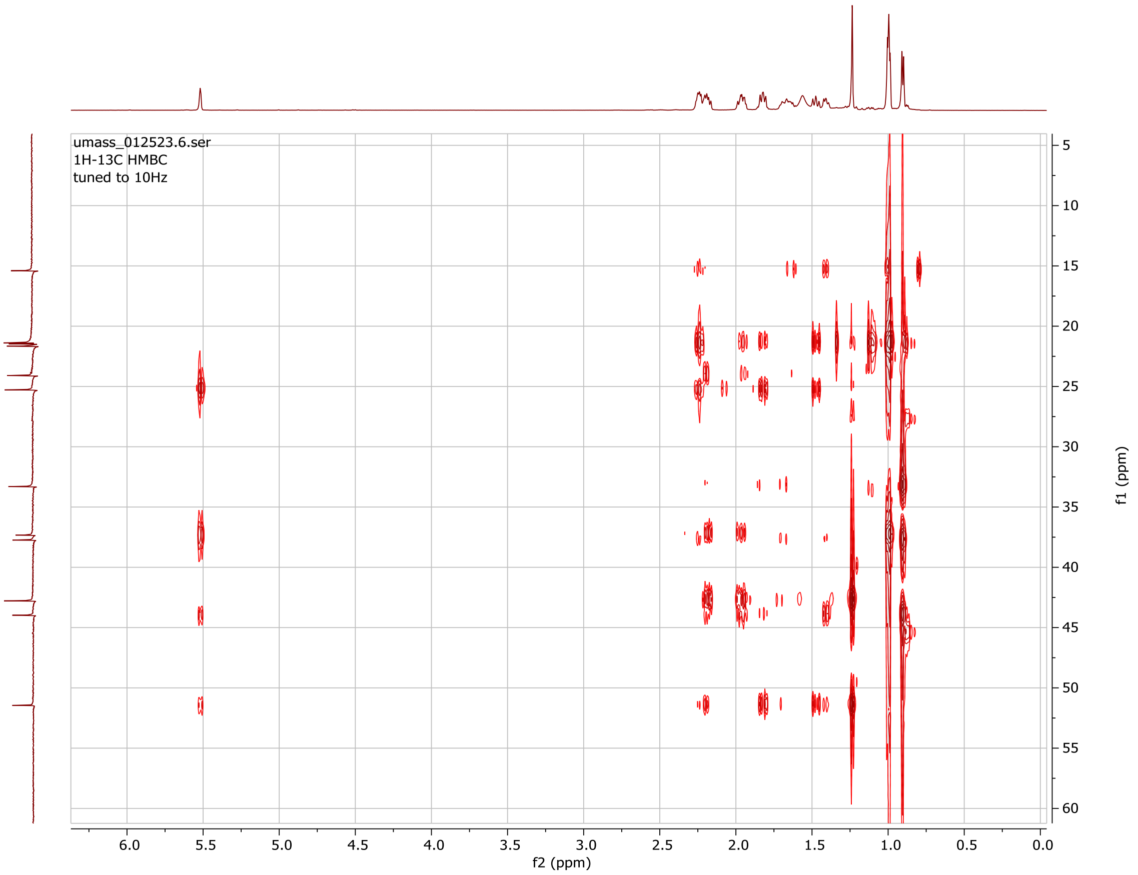

Figure S21b: HMBC spectrum of compound **9** (upfield)

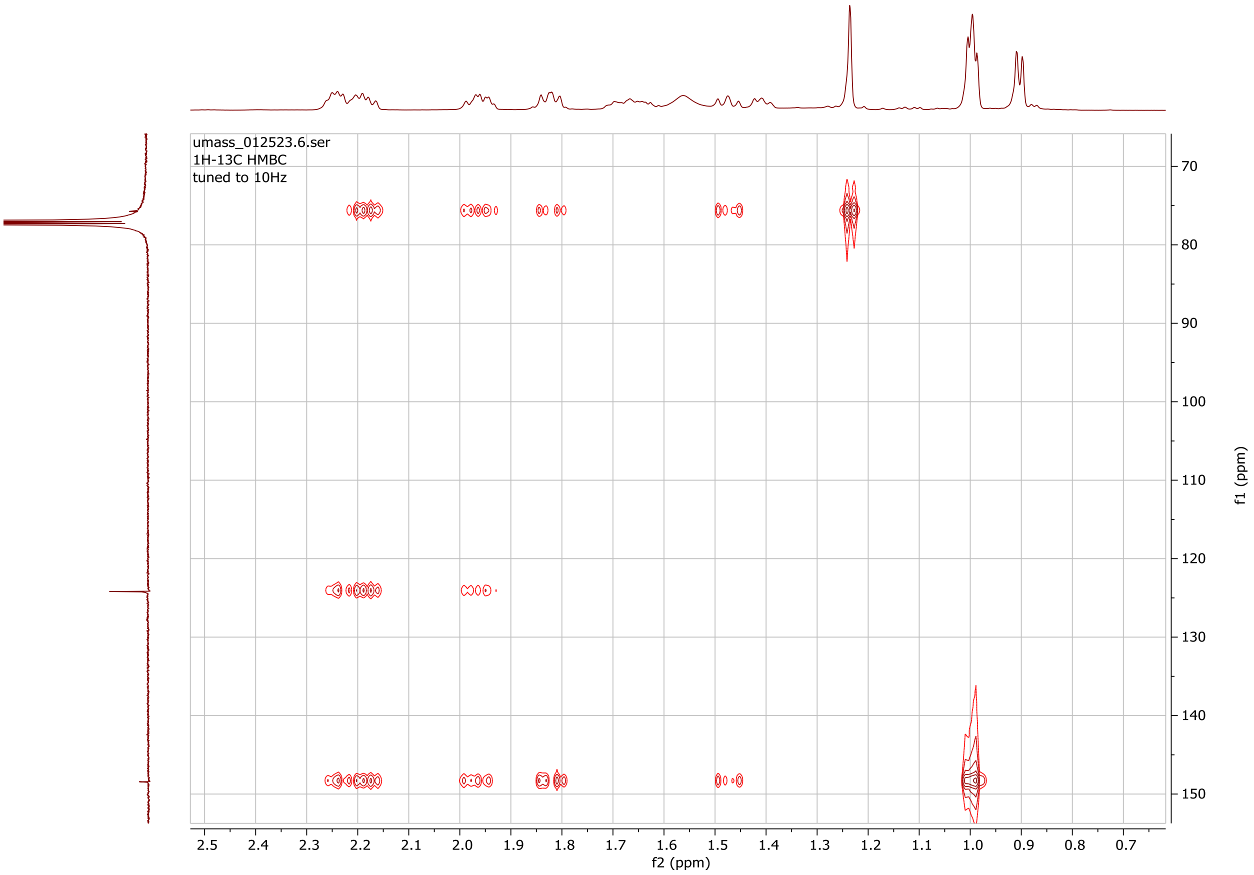

Figure S21c: HMBC spectrum of compound **9** (downfield)

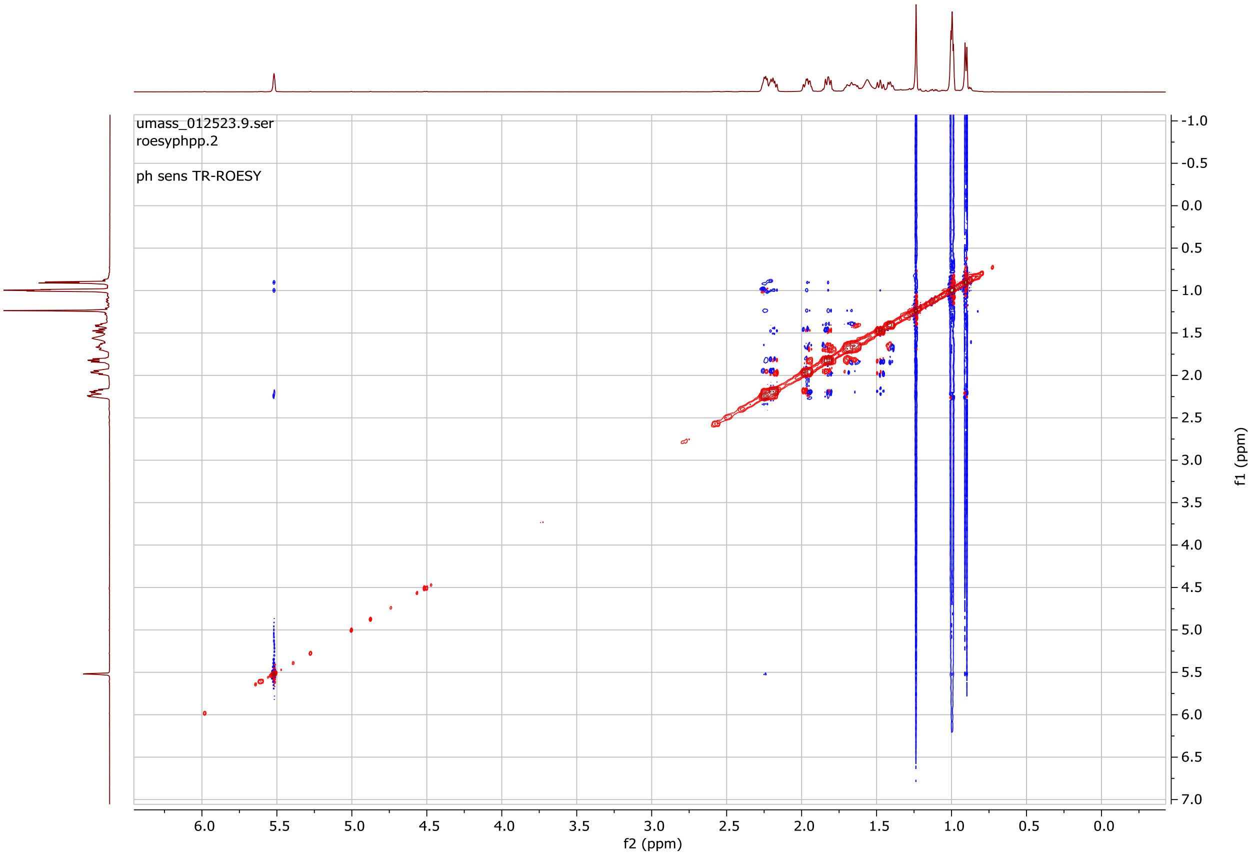

Figure S22a: ^1H-^1H ROESY spectrum of compound **9** (full spectrum)

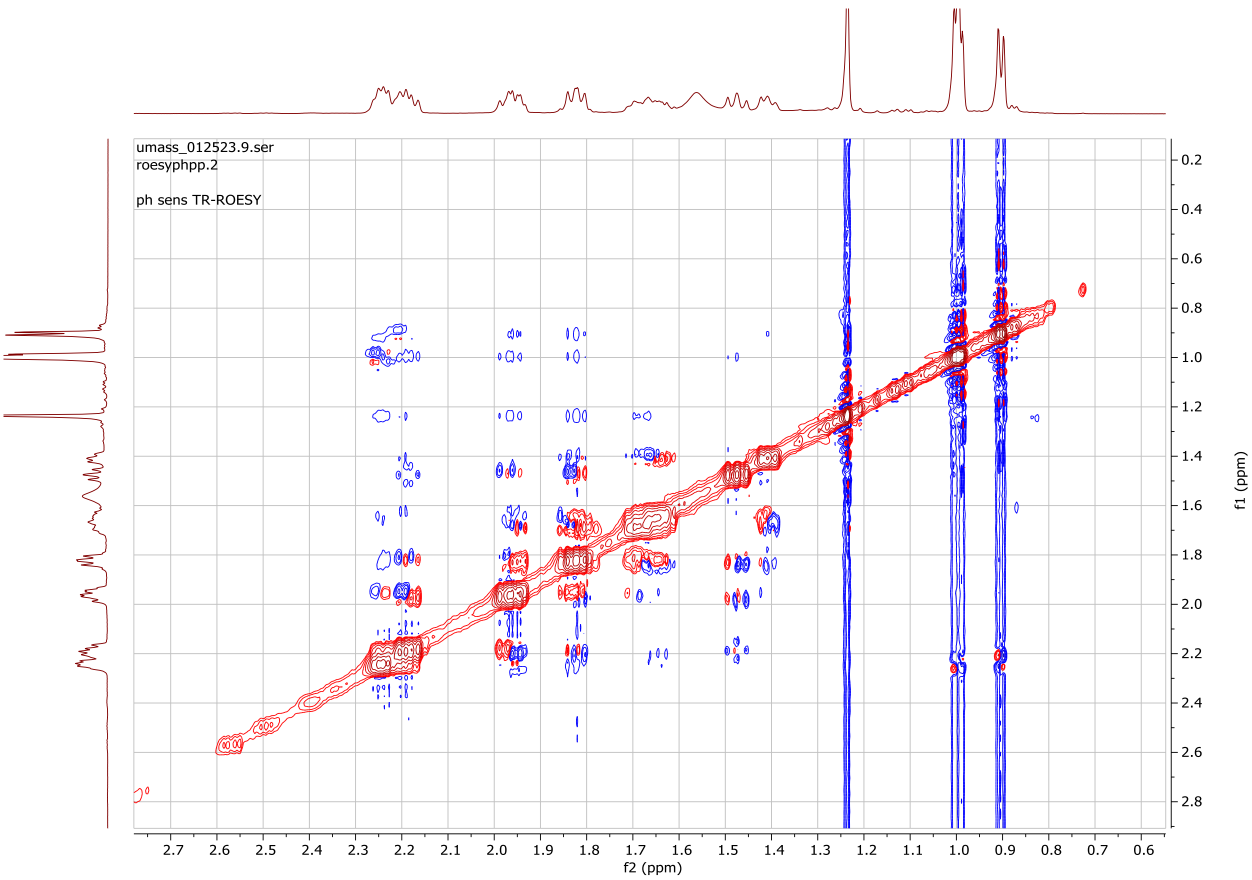

Figure S22b: ^{1}H-^{1}H ROESY spectrum of compound **9** (upfield)

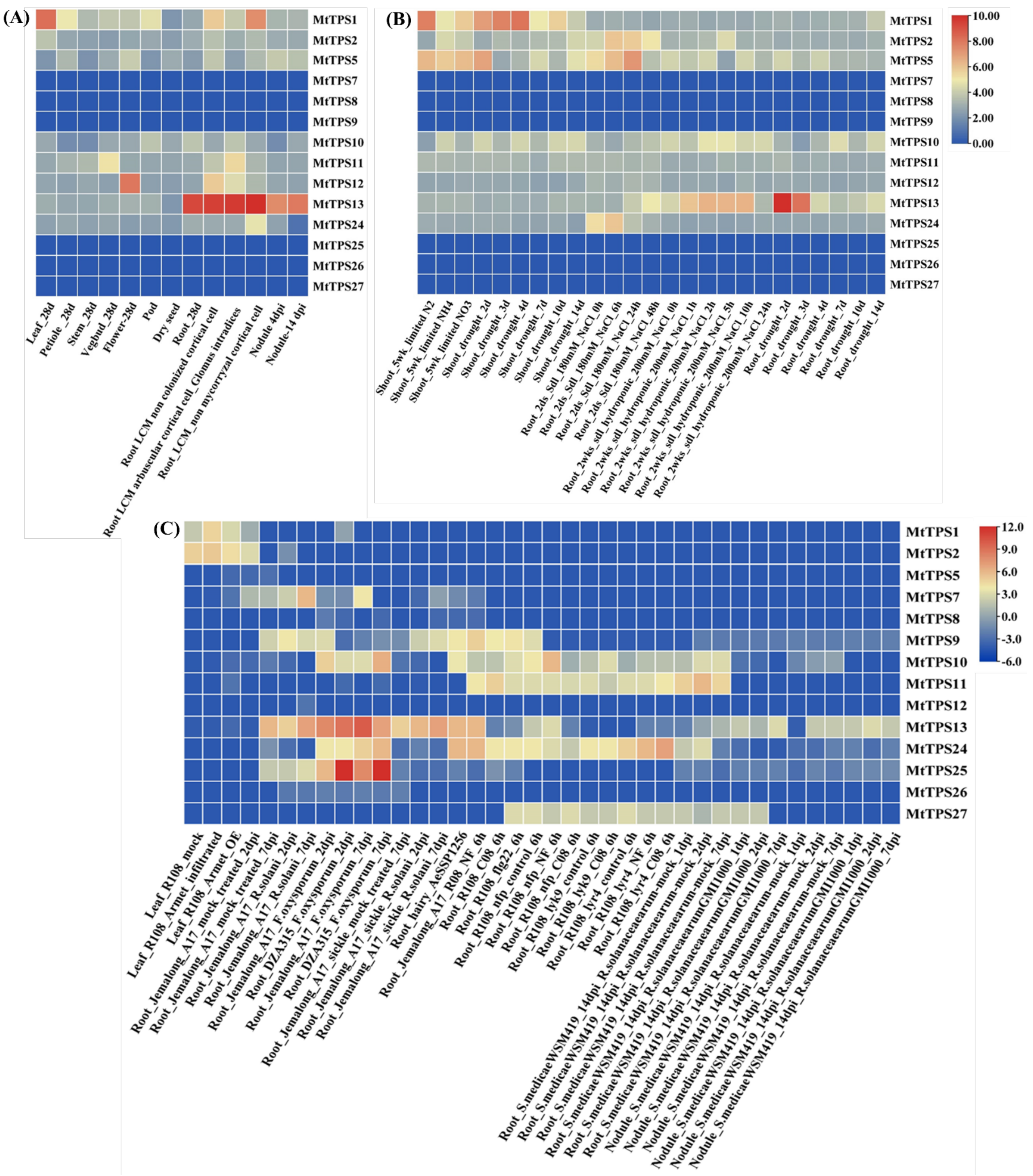

Figure S23. Heatmap showing differential gene expression of TPS-a subfamily members in response to various (a)biotic stresses.

Tissue-specific expression of TPS-a subfamily member (**A**) in different tissues (**B**) under different abiotic stress and (**C**) biotic stress expression of *Medicago truncatula.*

Robust multichip average (RMA) normalized data were collected from Noble Research Institute Legacy Gene Atlas V3 dataset by LIPME Team (https://medicago.toulouse.inrae.fr/MtExpress). Heatmap was generated by TBtools software based on the expression data. The color scale represents relative expression levels, with red indicating high expression levels and blue indicating lower expression levels.

Explanation of X-axis parameters.

- *Medicago truncatula* ecotpyes – cv. Jemalong A17; R108; DZA35
- Armet is an aphid effector protein which was infiltrated in leaf of *M. truncatula.*
- *Ae*SSP1256 proteins effectors secreted by *Aphanomyces euteiches*
- Pathogenic fungi *Rhizoctonia solani*; *Fusarium oxysporum*; *Ralstonia solanacearum* (GM11000)
- Pathogenic bacteria *Sinorhizobium medicae* (WSM419)
